# Stimulant medications affect arousal and reward, not attention networks

**DOI:** 10.1016/j.cell.2025.11.039

**Published:** 2025-12-24

**Authors:** Benjamin P. Kay, Muriah D. Wheelock, Joshua S. Siegel, Ryan V. Raut, Roselyne J. Chauvin, Athanasia Metoki, Aishwarya Rajesh, Andrew Eck, Jim Pollaro, Anxu Wang, Vahdeta Suljic, Babatunde Adeyemo, Noah J. Baden, Kristen M. Scheidter, Julia S. Monk, Forrest I. Whiting, Nadeshka Ramirez-Perez, Samuel R. Krimmel, Russell T. Shinohara, Brenden Tervo-Clemmens, Robert J.M. Hermosillo, Steven M. Nelson, Timothy J. Hendrickson, Thomas Madison, Lucille A. Moore, Óscar Miranda-Domínguez, Anita Randolph, Eric Feczko, Jarod L. Roland, Ginger E. Nicol, Timothy O. Laumann, Scott Marek, Evan M. Gordon, Marcus E. Raichle, Deanna M. Barch, Damien A. Fair, Nico U.F. Dosenbach

**Affiliations:** 1 Department of Neurology, Washington University School of Medicine, St. Louis, MO, USA; 2 Allied Labs for Imaging Guided Neurotherapies (ALIGN), Washington University School of Medicine, St. Louis, MO, USA; 3 Mallinckrodt Institute of Radiology, Washington University School of Medicine, St. Louis, MO, USA; 4 Department of Psychiatry, New York University Langone Center for Psychedelic Medicine, New York, NY, USA; 5 Allen Institute, Seattle, WA, USA; 6 Department of Physiology & Biophysics, University of Washington, Seattle, WA, USA; 7 Taylor Family Department of Neurosurgery, Washington University School of Medicine, St Louis, MO, USA; 8 Penn Statistics in Imaging and Visualization Center, Perelman School of Medicine, Philadelphia, PA, USA; 9 Department of Biostatistics, Epidemiology and Informatics, Perelman School of Medicine, Philadelphia, PA, USA; 10 Center for AI and Data Science for Integrated Diagnostics, Perelman School of Medicine, Philadelphia, PA, USA; 11 Department of Psychiatry and Behavioral Sciences, University of Minnesota, Minneapolis, MN, USA; 12 Masonic Institute for the Developing Brain, University of Minnesota Medical School, Minneapolis, MN, USA; 13 Department of Pediatrics, University of Minnesota, Minneapolis, MN, USA; 14 Minnesota Supercomputing Institute, University of Minnesota, Minneapolis, MN, USA; 15 Department of Psychiatry, Washington University School of Medicine, St. Louis, MO, USA; 16 Department of Biomedical Engineering, Washington University in St. Louis, St. Louis, MO, USA; 17 Department of Psychological and Brain Sciences, Washington University in St. Louis, St. Louis, MO, USA; 18 Institute of Child Development, University of Minnesota, Minneapolis, MN, USA; 19 Program in Occupational Therapy, Washington University in St. Louis, St. Louis, MO, USA; 20 Department of Pediatrics, Washington University School of Medicine, St. Louis, MO, USA; 21 Lead contact

## Abstract

Prescription stimulants (e.g., methylphenidate) are thought to improve attention, but evidence from prior fMRI studies is conflicted. We utilized resting-state fMRI data from the Adolescent Brain Cognitive Development Study (*n* = 11,875; 8–11 years old) and validated the functional connectivity findings in a precision imaging drug trial with highly sampled (*n* = 5, 165–210 min each) healthy adults (methylphenidate 40 mg). Stimulant-related connectivity differences in sensorimotor regions matched fMRI patterns of daytime arousal, sleeping longer at night, and norepinephrine transporter expression. Taking stimulants reversed the effects of sleep deprivation on connectivity and school grades. Connectivity was also changed in salience and parietal memory networks, which are important for dopamine-mediated, reward-motivated learning, but not the brain’s attention systems (e.g., dorsal attention network). The combined noradrenergic and dopaminergic effects of stimulants may drive brain organization towards a more wakeful and rewarded configuration, improving task effort and persistence without effects on attention networks.

## In brief

Stimulant medications (e.g., methylphenidate) were thought to improve attention by acting on the brain’s attention networks. Functional connectivity data now reveal that stimulants are associated with changes in arousal and reward, but not attention systems, suggesting that they enhance performance by increasing vigilance and perceived task value, not attentional capabilities.

## Graphical abstract

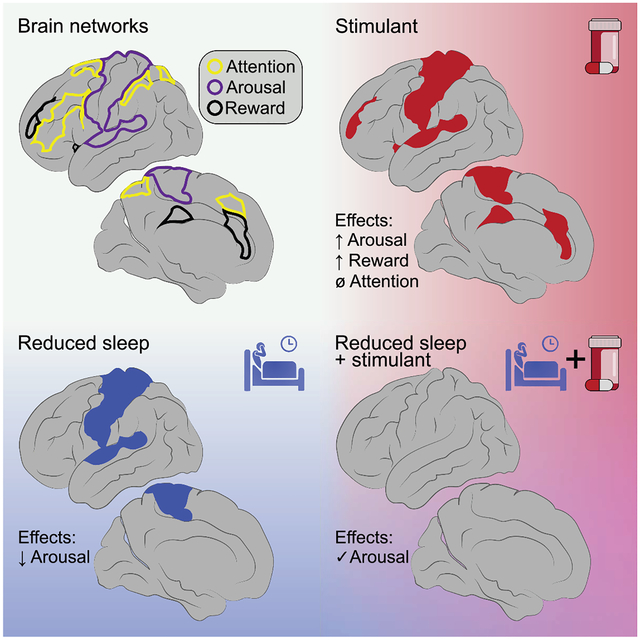

## INTRODUCTION

Methylphenidate, lisdexamfetamine, and other prescription stimulants are thought to be potent wakefulness- and attention-promoting^1^ norepinephrine and dopamine reuptake inhibitors^2,3^ used by 6.1% of Americans across all ages (and up to 24.6% of boys ages 10–19)^4,5^ for attention deficit hyperactivity disorder (ADHD),^6^ traumatic brain injury (TBI),^7^ narcolepsy,^8,9^ and depression^10^; as appetite suppressants,^11–14^ cognitive enhancers (nootropics),^15–17^ and drugs of abuse^18^; and to enhance athletic performance.^19^ The wakefulness-promoting properties of amphetamine were discovered in 1929, and it was later prescribed for narcolepsy and used by soldiers in World War II.^1^ Charles Bradley discovered that amphetamine seemed to treat what he termed “behavioral problem children” in 1937,^20^ although stimulants were not widely prescribed for behavior until the 1970s, and the term ADHD was not widely used until 1980.^6^ Bradley proposed that stimulants might act on attention and impulsivity by enhancing the activity of attention-promoting brain regions to increase voluntary control over action.^20^ Early research identified regions in prefrontal cortex associated with voluntary allocation of attention as being modulated by stimulants through frontostriatal circuits,^21–27^ while the current understanding has evolved to include more diverse brain systems, including sensorimotor and salience regions that serve a facilitatory role in attention.^23,28,29^ The relative effect of stimulants on these brain systems remains unclear.

The belief that stimulants act primarily on prefrontal cortex, along with evidence of beneficial effects on tasks involving attention and working memory in rodents,^30–32^ primates,^33^ and humans,^34–37^ led to the popular belief that stimulants improve attentional ability or perhaps even cognitive ability in general.^15–17^ However, closer examination of behavioral experiments shows that performance follows an inverted U-shaped curve.^32,38,39^ Lower performers improve the most with stimulants, while high-performers do not improve,^30,32,33,36,37^ or even perform worse,^40^ but mistakenly perceive their performance as improved.^41^ The most consistent behavioral effects of stimulants are improved reaction time,^31,36^ time discrimination,^36^ premature responses/impulsivity,^32,33^ distractor suppression,^42^ effort,^40^ persistence,^34,35^ and motivation.^37,43,44^

Task-based fMRI studies have shown stimulant effects in prefrontal cortex as well as disparate brain regions whose functions are difficult to reconcile, e.g., insula, supplemental motor area, and thalamus.^36^ One challenge in interpreting task-fMRI results in the context of stimulants is that brain activity selectively evoked by the task contrast can be confounded by stimulant-driven differences in task performance.^45^ Resting-state fMRI (rs-fMRI) functional connectivity (FC)^46^ is not subject to performance confounds and provides a conceptual framework for synthesizing regional results into network-level hypotheses.^47–50^ While a growing number of studies have leveraged rs-fMRI to study the neural correlates of stimulants, no coherent mechanistic hypothesis of medication effects has emerged.^51^

Many prior human rs-fMRI studies^24,25,27,52,53^ and some functional connectivity studies of task-fMRI data^54^ reported significant FC changes associated with stimulants in the dorsal and ventral attention networks (DAN, VAN)^55–59^ and in cognitive control networks such as the frontoparietal network (FPN)^60–63^ and the default mode network (DMN),^64,65^ which intersect prefrontal cortex. However, these findings of stimulant-related changes in attention and control networks were not replicated in larger studies.^66,67^ Some rs-fMRI^24,25,28,52,68^ and positron emission tomography (PET)^68,69^ studies noted changes in primary sensorimotor regions associated with stimulants,^52^ attention,^28^ and ADHD^23^ that “may be unexpected given the traditional view of ADHD as primarily involving executive control regions and networks.”^28^ Other studies^70–72^ reported stimulants affecting FC of the salience network (SAL), which is thought to govern reward- and aversion-motivated behavior.^73–75^ In several studies,^27,54,70,71,76,77^ the reported default mode or salience regions may have included portions of the parietal memory network (PMN), which is closely related to SAL^78–80^ by shared dopaminergic connections^81–83^ with the nucleus accumbens^37,84^ and provides memory for goal-directed actions.^85–87^ Thus, stimulants may modulate cognition through multiple brain mechanisms, the relative roles of which remain incompletely understood^51^ with conflicting results from human neuroimaging studies.^24–28,52,53,66,67,69–72,76,77,88,89^

Prior human neuroimaging studies have involved sample sizes from *n* = 10^69^ to *n* = 99^66^ participants taking stimulants with relatively brief (6^26,28^ to 24 min^90^) fMRI acquisitions subject to reliability concerns,^91–95^ and few attempted to replicate their results in independent data or with complementary designs.^68,71^ Recent work has shown more reliable results are achieved with thousands of participants for brain-wide association studies (BWAS),^96^ extended-duration repeated fMRI scans for precision functional mapping (PFM) studies,^92,94,97–100^ and the use of discovery and replication sets for validation.^97,98,101^ Many prior analyses used a region of interest (ROI) approach,^24,25,27,52,53,66,70–72^ whereas advances in computational methods have now enabled data-driven approaches with increased statistical power.^102–104^ Prior imaging studies did not control for the effects of sleep, even though inadequate sleep (less than 9 h of sleep per night in children)^105^ is common^106,107^ and associated with cognitive decrements.^108^

The unexpected^28^ relationship between stimulants and primary sensorimotor cortex has been interpreted as relating to inhibition of motoric output in hyperactive individuals^23,88^ based on the observation that ADHD is associated with decreased primary motor cortex short interval cortical inhibition in behavioral,^109^ fMRI,^110^ and transcranial magnetic stimulation (TMS) studies.^88,111–114^ However, recent findings in functional neuroanatomy and connectomics provide an alternative context in which to interpret stimulant-related differences in motor cortex. Multi-modal precision imaging research has shown that primary motor cortex is not a simple homunculus, but is interleaved with the somato-cognitive action network (SCAN)^115^ with diverse functions including regulation of sympathetic outflow.^116^ Interleaved action^117^ and motor regions reflect arousal state^118–120^ such that FC within sensorimotor (SM), auditory (AUD), and visual (VIS) networks is increased during sleep and decreased during wakefulness.^92,121–124^

In this study, we used rs-fMRI data from the Adolescent Brain Cognitive Development (ABCD) Study (*n* = 11,875).^125,126^ We employed a data-driven whole-connectome strategy to model differences in FC in attention, arousal, and salience/memory networks related to prescription stimulants without *a priori* exclusion of other networks. Network level analysis (NLA)^127,128^ was used to account for multiple comparisons. The findings were validated^97,98^ with a precision imaging drug trial (PIDT) of methylphenidate 40 mg in *n* = 5 healthy adults without ADHD (165–210 min, mean 186 min, of rs-fMRI data per participant).^129^

## RESULTS

### Stimulant use is prevalent in children

In the ABCD Study (*n* = 11,875, 8–11 years old, data collected 2016–2019), 7.8% of children (74.6% boys) were prescribed a stimulant and 6.2% (74.0% boys) took the stimulant on the morning of their MRI scans. Data on specific dose and formulation were not available in the ABCD Study. Using stringent criteria,^130^ 3.7% of children (69.4% boys) had ADHD, of whom 42.7% were prescribed a stimulant and 34.9% took the stimulant on the morning of scanning. Only 20.7% of children who took a stimulant on the morning of scanning met criteria for ADHD. Using less stringent criteria for identifying ADHD (see STAR Methods), 76.2% of children taking a stimulant had ADHD. A sample of *n* = 5,795 children with complete data, including sufficient low-motion fMRI, included *n* = 337 (73.0% boys) children taking a stimulant on the morning of their scans.

### Stimulants change children’s action, motor, and salience connectivity

**Figure 1.**
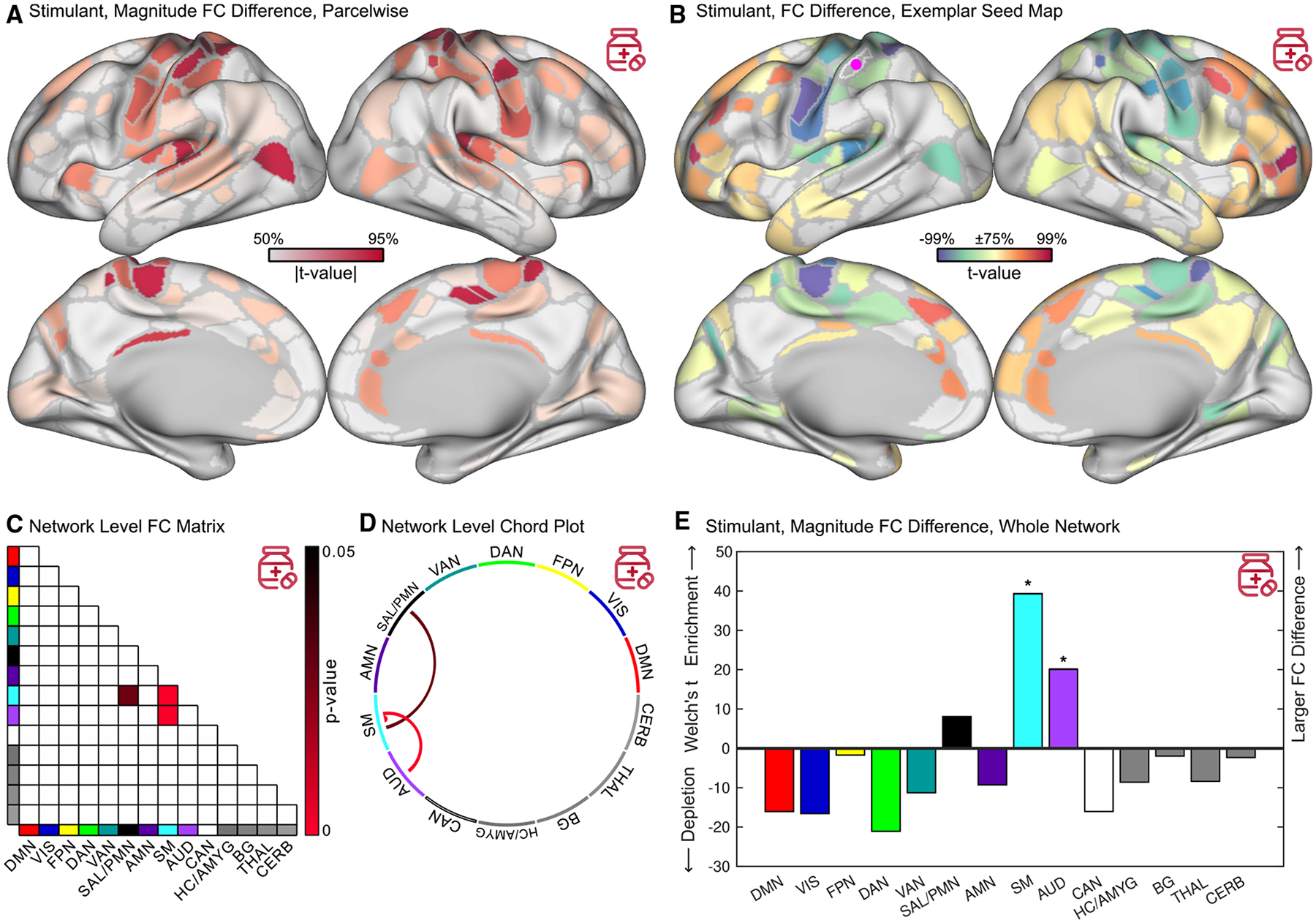
Stimulant-related functional connectivity differences ABCD Study data, *n* = 5,795 children, *n* = 337 taking a stimulant. Stimulant-related findings are color-coded red. (A) Magnitude (root-mean-square) of functional connectivity (FC) difference shown on the Gordon-Laumann cortical parcellation.^133^ The color scale is thresholded between the 50^th^ and 95^th^ percentiles to facilitate visual comparison between figures. (B) Differences in FC with an exemplar (most affected by stimulants) seed parcel in the motor-hand region (purple dot). (C and D) Significant (FWER *p* < 0.05) differences in FC between network pairs using network level analysis (NLA). (E) Magnitude (NLA, Welch’s *t*-statistic) of FC differences in whole networks relative to the whole connectome. Significant (FWER *p* < 0.05) differences are indicated by a *. DMN, default mode; VIS, visual; FPN, fronto-parietal; DAN, dorsal attention; VAN, ventral attention; SAL, salience; PMN, parietal memory; AMN, action-mode; SM, somato-cognitive action/motor; AUD, auditory; CAN, context association; HC, hippocampus; AMYG, amygdala; BG, basal ganglia; THAL, thalamus; CERB, cerebellum. See also Figures S1–S8.

To visualize the relative FC differences (*t*-values; see STAR Methods for covariates) associated with pre-scan stimulant use in each brain region, we computed the magnitude of FC differences over the edges connected to each region. The largest stimulant-related FC differences were in somato-cognitive action, primary motor, auditory, salience, and parietal memory regions (Figure 1A). An exemplar parcel-wise seed map using a motor-hand parcel with the greatest FC difference is shown in Figure 1B. For the full FC matrix and effect sizes, see Figure S2. The pattern of FC differences modeled using linear covariates was the same as for a sub-cohort matched for sample size and demographic characteristics (Figure S2). The nucleus accumbens is thought to be central to dopamine-mediated processing of reward, salience, and effort.^131,132^ An additional nucleus accumbens seed map showed high FC with canonical salience regions in cortex (e.g., anterior inferior right insula)^73,75^ but no significant difference related to stimulants (Figure S3).

The somato-cognitive action and sensorimotor networks, which are interleaved along the central sulcus, were treated as one sensorimotor (SM) network for the purpose of statistical comparisons (Figure S1). Among pairs of canonical networks, stimulants were associated with significantly decreased FC within and between SM and AUD networks (NLA, Westfall-Young step-down family-wise error rate [FWER]-corrected^134^ *p* < 0.05). Stimulants were associated with significantly increased FC between SM and SAL/PMN (Figures 1C and 1D). Among all edges within and between each network, stimulants were associated with the largest differences in FC in SM and AUD (FWER, *p* < 0.05) and a trend toward relatively larger FC differences in SAL/PMN (Figure 1E). The 98^th^ percentile (across edges) effect sizes (beta values) were −0.030 (Cohen’s *d* = −0.17) for stimulants and −0.011 (Cohen’s *d* = −0.064) for sleep. There were no significant FC differences in attention (DAN, VAN) or control (FPN) networks, despite 95% power to detect stimulant-related differences in attention networks (Table 1).

It has been hypothesized that children with ADHD may show different changes in FC in response to stimulant intervention during an attention-demanding task compared to rest.^54^ The n-back task was used in the ABCD Study to engage working memory and cognitive control in adolescents.^135^ fMRI data from the n-back task was treated as rest and analyzed without regressing out the task paradigm. Stimulant-related differences in n-back FC were parcel-wise highly correlated with those of resting FC (*r* = 0.45, spin test^136,137^ *p* = 0.0015) (Figure S4). Stimulants were not associated with significant differences in task-evoked fMRI activation for 0-back or 2-back vs. fixation, (Figure S4), although power may have been limited by fewer children with high-quality n-back data (*n* = 109 taking stimulants) and technical issues specific to task design in the ABCD Study.^138^

Differences in the precise molecular action of different stimulant drugs have been reported.^3^ An analysis of the ABCD data separating stimulants into specific drugs (methylphenidate, lisdexamfetamine, etc.) showed the same pattern of FC differences for each drug (see Figure S5 for a breakdown of stimulants by active ingredient). The stimulant-related patten of FC differences was not observed for cetirizine, a common allergy medication taken by *n* = 291 children on the day of scanning that is not psychoactive and was therefore chosen as a negative control.^139^ The cetirizine-related differences in FC, which were below the threshold for significance, were parcel-wise not correlated with those of stimulant on the cortex (*r* = 0.059, spin test *p* = 0.39) (Figure S5).

Stimulant-related FC differences were specifically associated with taking the stimulant drug on the morning of scanning. The subset of children (*n* = 76) who were prescribed stimulants but did not take them on the morning of scanning showed no significant FC differences compared to *n* = 5,382 children not prescribed or taking a stimulant. Conversely, comparing the 337 children taking a stimulant on the day of scanning to the 76 children who did not take their stimulant on the day of scanning reproduced the differences in FC seen when comparing stimulants on the day of scanning to no stimulants at all (Figure S6). Results were not due to differences in ADHD diagnosis or head motion (Figures S6 and S7; Table S1).

### Stimulant-driven connectivity changes were validated in an adult trial

**Figure 2.**
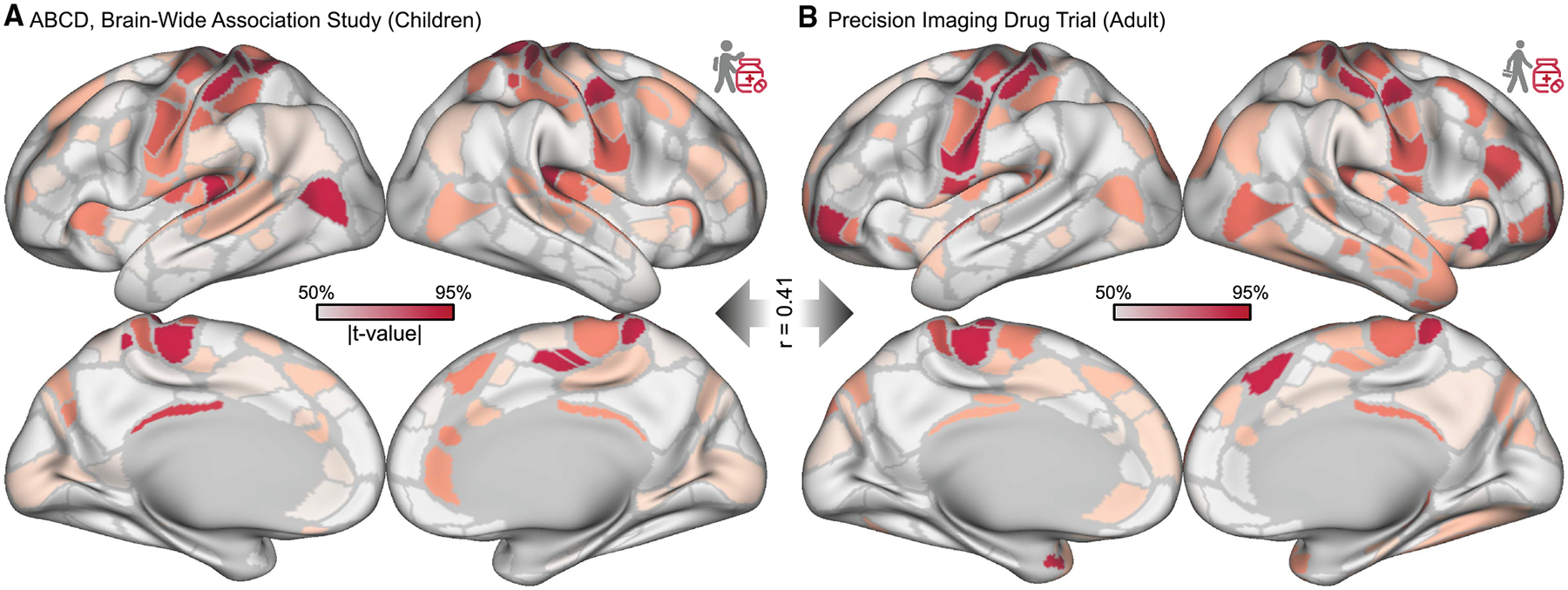
Stimulant effects validated in precision imaging drug trial (A) Magnitude (root-mean-square) of functional connectivity (FC) differences shown on the Gordon-Laumann cortical parcellation^133^ for 337 children taking stimulants in the ABCD Study (total *n* = 5,795). The color scale is thresholded between the 50^th^ and 95^th^ percentiles to facilitate visual comparison. (B) Magnitude of acute FC differences in adult participants (*n* = 5) given methylphenidate 40 mg in a controlled study. The cortical maps are correlated at *r* = 0.41 (spin test *p* < 0.001). See also Figure S8.

The ABCD Study does not experimentally control for why children take stimulants. Therefore, differences in FC associated with stimulants were validated^97,98^ in a trial with 5 healthy adult participants (165–210 min of rs-fMRI data each). Each participant had 120–180 min of rs-fMRI data off stimulants and 15–60 min of rs-fMRI data on methylphenidate (Ritalin) 40 mg.^129^ The study design controlled for factors correlated with stimulant use (e.g., ADHD diagnosis) by recruiting participants who were not prescribed a stimulant and comparing FC within the same individuals on and off stimulants. The largest stimulant-related changes in FC in these controlled data were the same as in the ABCD Study—decreased within-network FC in SM (mixed effects *p* value = 0.008) and increased cross-network FC between SM and SAL/PMN (*p* = 0.013). For parcel-wise correlation between the two studies’ cortical magnitude FC difference maps (*r* = 0.41, spin test *p* < 0.0001), see Figure 2. For edgewise correlation between the two studies, see Figure S8.

### Stimulants mimic the effects of getting more sleep

The greatest stimulant-related differences in FC were in somato-cognitive action and motor networks associated with arousal/wakefulness^118–124^; therefore, we characterized the FC pattern associated with getting more sleep and compared it to the FC pattern associated with taking stimulants. Parents of children in the ABCD Study were asked, “How many hours of sleep does your child get on most nights?”^140^ Parent-reported sleep duration served as a surrogate measure of being better rested, or arousal/wakefulness, at the time of scanning.

**Figure 3.**
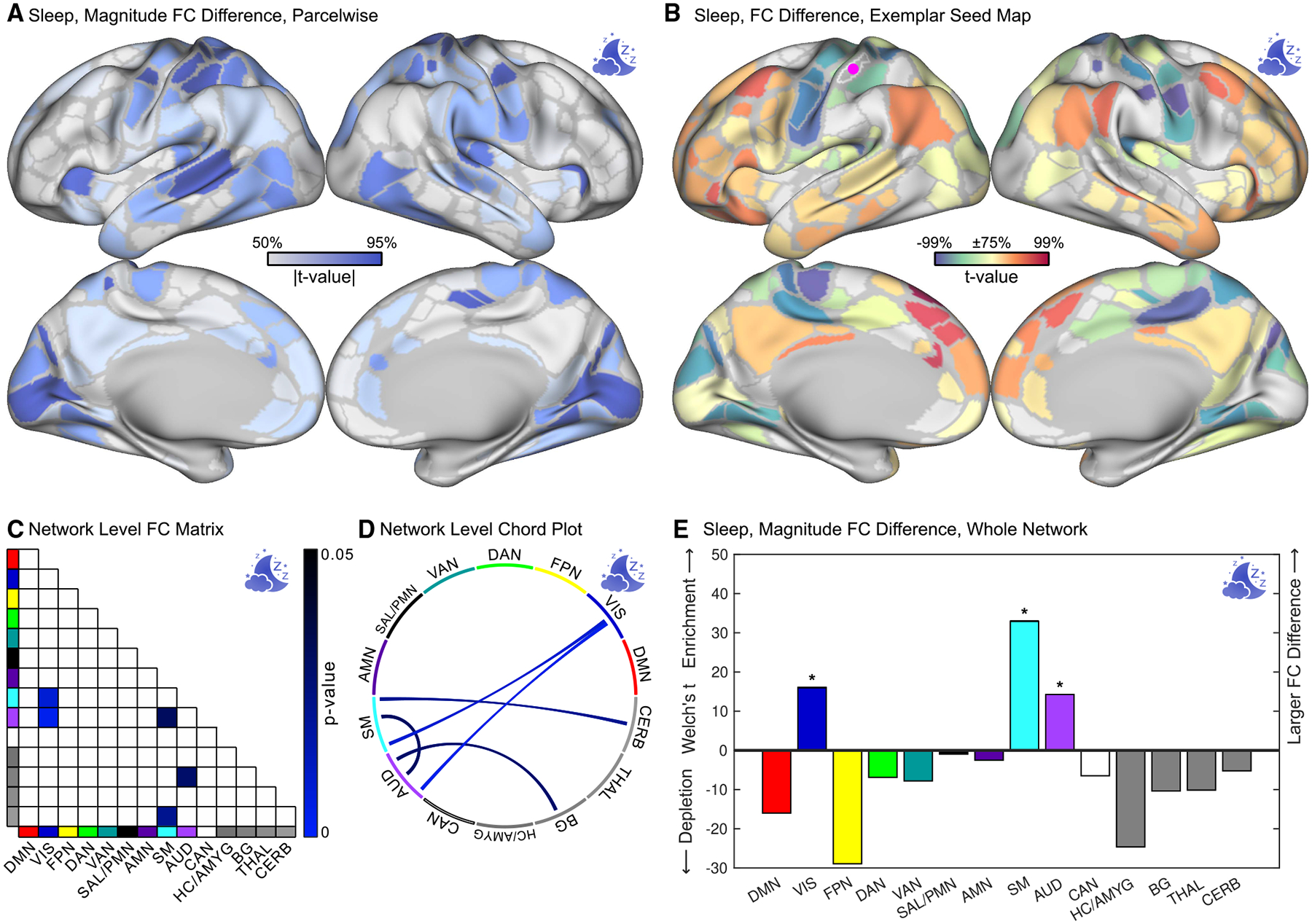
Sleep-duration-related functional connectivity differences ABCD Study data, *n* = 5,795 children. Sleep-related findings are color-coded blue. (A) Magnitude (root-mean-square) of functional connectivity (FC) differences shown on the Gordon-Laumann cortical parcellation.^133^ The color scale is thresholded between the 50^th^ and 95^th^ percentiles to facilitate visual comparison between figures. (B) Differences in FC with an exemplar seed parcel in the somatomotor hand region (purple dot). (C and D) Significant (FWER *p* < 0.05) differences in FC between network pairs using NLA. (E) Magnitude (Welch’s *t*-statistic) of FC difference in whole networks relative to the whole connectome. Significant (NLA, FWER *p* < 0.05) changes are indicated by a *. DMN, default mode; VIS, visual; FPN, fronto-parietal; DAN, dorsal attention; VAN, ventral attention; SAL, salience; PMN, parietal memory; AMN, action-mode; SM, somato-cognitive action/motor; AUD, auditory; CAN, context association; HC, hippocampus; AMYG, amygdala; BG, basal ganglia; THAL, thalamus; CERB, cerebellum. See also Figures S1, S2, S7, and S9.

Longer sleep duration was associated with FC differences in motor, auditory, and visual regions in a pattern similar (cortex + subcortex *r* = 0.58; cortex-only *r* = 0.58; spin test *p* < 0.0001) to that of taking a stimulant (Figure 3A). Sleep duration was also extremely similar to stimulants in the exemplar parcel-wise seed map (cortex + subcortex *r* = 0.87; cortex-only *r* = 0.86; spin test *p* < 0.0001) (Figure 3B; see Figure S2 for the full FC matrix). At the level of network pairs, sleep duration was associated with significantly (FWER *p* < 0.05) decreased FC within SM and decreased FC between SM and primary sensory networks (AUD and VIS) (Figures 3C and 3D). At the level of whole networks, sleep duration was associated with significant (FWER *p* < 0.05) changes in SM, AUD, and VIS. Thus, while stimulant-and sleep-related patterns of FC were similar, stimulants were associated with greater relative differences involving SAL/PMN than sleep duration.

### Arousal regions show the strongest sleep-related connectivity differences

To further validate our finding of decreased arousal-related FC within SM, AUD, and VIS, we used data from three independent extramodal studies. The EEG alpha slow wave index (alpha/delta power ratio)^141^ is an instantaneous measure of arousal state.^142,143^ Falahpour, Goodale et al. generated a template map for detecting arousal by recording simultaneous EEG and fMRI in a study of *n* = 10 adults.^123,124^ Variability in respiratory rate is also correlated with moment-to-moment fluctuations in arousal and was employed by Raut et al. to generate a map of arousal^119^ from *n* = 190 participants with real-time respiratory data in the Human Connectome Project.^144^ Stimulants increase synaptic levels of norepinephrine,^2,3^ a neurotransmitter strongly associated with arousal.^145^ Hesse et al. generated a map of norepinephrine transporter (NET) density using PET (*n* = 20).^146,147^

**Figure 4.**
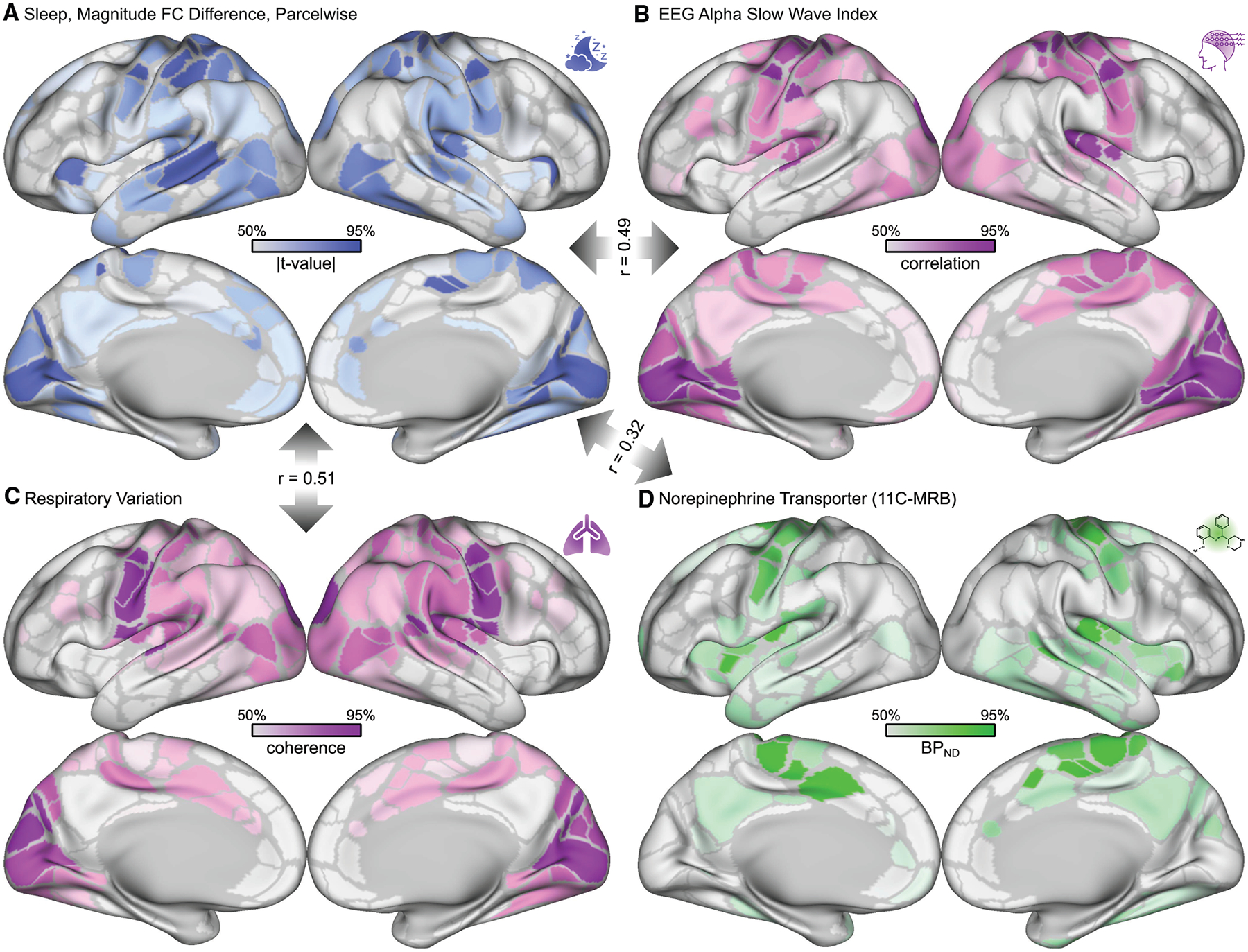
Sleep duration effects validated against independent brain maps of arousal (A) Magnitude (root-mean-square) of functional connectivity (FC) differences related to sleep duration shown on the Gordon-Laumann cortical parcellation^133^ (ABCD Study, *n* = 5,795). The color scale is thresholded between the 50^th^ and 95^th^ percentiles to facilitate visual comparison. (B) Arousal template obtained by correlating EEG alpha slow wave index (alpha/delta power ratio) with fMRI signal intensity (*n* = 10).^123,124^ (C) Arousal map obtained from coherence between respiratory variation and fMRI signal intensity based on Human Connectome Project (*n* = 190).^119^ (D) Non-displaceable binding potential for 11C-MRB (methylreboxetine) in a positron emission tomography (PET) study (*n* = 20).^146,147^ Correlations between cortical maps are shown in gray arrows and summarized in Table S2. The correlation between the EEG- and respiration-derived arousal maps was *r* = 0.60 (spin test *p* < 0.0001). See also Figure S10.

We compared the FC differences related to sleep duration in the ABCD Study with each of these three independent arousal-related brain maps. Sleep was correlated with the EEG alpha slow wave index-fMRI map^123,124^ at *r* = 0.49 (spin test *p* < 0.0001) (Figure 4B; Table S2). Sleep was correlated with the respiratory variation-fMRI map^119^ at *r* = 0.51 (spin test *p* = 0.0015) (Figure 4C). Sleep was correlated with the PET norepinephrine transporter density map^146,147^ at *r* = 0.32 (spin test *p* = 0.005) in cortical parcels (Figure 4D; Table S2). Receptor density maps for dopamine, which is modulated by stimulants but less strongly associated with arousal,^145^ are shown in Figure S10. Stimulant-related FC differences were also significantly (spin test *p* < 0.05) correlated with maps of arousal and norepinephrine receptor density (Table S2).

### Stimulants and sleep have similarly beneficial effects on performance

Stimulants^34,35^ and getting sufficient sleep^148,149^ are both thought to have beneficial effects on attention and working memory. The ABCD Study collected data on parent-reported school letter grade, out-of-scanner performance on the NIH Toolbox,^150^ and in-scanner performance on the n-back task. These cognitive measures were modeled against stimulants taken on the day of scanning and sleep duration with age, sex, and socioeconomic covariates (STAR Methods). ADHD was associated with significantly worse school grades, NIH Toolbox performance, and rate of correct responses on the n-back, while getting more sleep was associated with significant improvement in all of these measures (Table 2). Children with ADHD who took a stimulant had improved cognitive performance on all measures compared to those who did not take a stimulant (significant ADHD × stimulant interaction), and children with less sleep had better school grades if they took a stimulant (significant negative stimulant × sleep interaction). Children getting adequate sleep who did not have ADHD did not have better school grades, NIH Toolbox scores, or rate of correct responses on the n-back compared to those who did not take a stimulant. Taking a stimulant did significantly improve reaction time on the n-back by about 100 ms independent of other factors. Thus, overall, stimulants improved cognitive performance only for participants with ADHD or insufficient sleep (see *p* values in Table 2).

### Stimulants rescue sleep-deficit-induced changes

Only 48% of children in the ABCD Study were reported by their parents as getting the recommended^105^ 9 or more hours of sleep per night. Taking stimulants and longer average sleep duration (being better rested) had similar effects on brain connectivity. Therefore, we performed subanalyses of the relations of sleep to behavior and FC in subsets of children taking and not taking stimulants. There was no significant association between taking a stimulant and sleep duration after accounting for ADHD diagnosis and demographic covariates (*p* = 0.23) (Figure S9). Behaviorally, children who slept longer (per parent report) had significantly better school grades, NIH Toolbox scores, and rate of correct responses on the n-back (Table 2). Conversely, children with less sleep had significant decrements in their cognitive performance. However, the deleterious association of sleep deprivation with cognitive performance was not significant in the subset of children taking stimulants (*n* = 337). Children getting less sleep but taking a stimulant (stimulant × −sleep interaction) received grades that were significantly better than those of children getting less sleep and not taking a stimulant and equal to the grades of well-rested children not taking a stimulant (Table 2).

**Figure 5.**
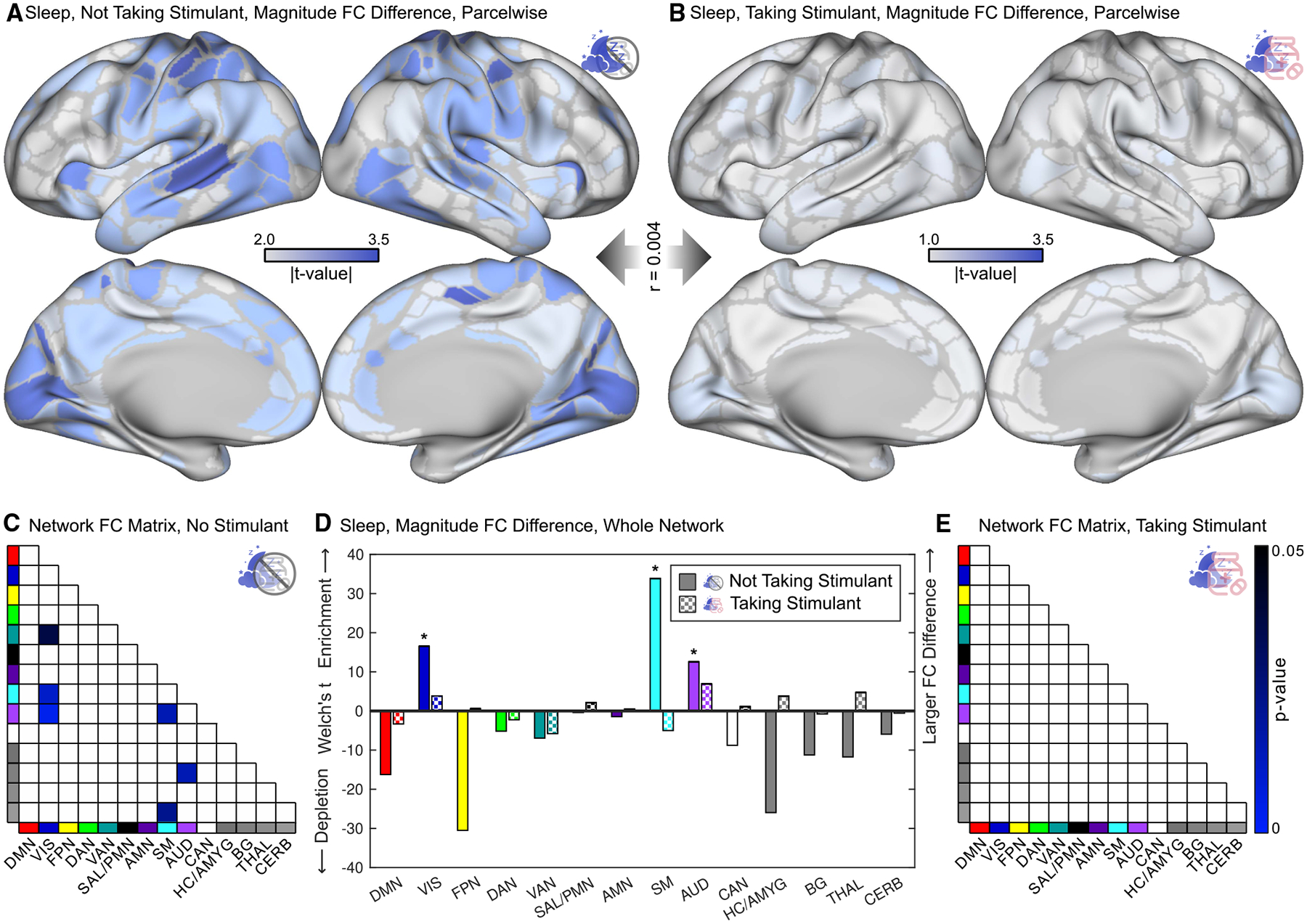
Sleep duration and stimulant use’s interacting brain effects ABCD Study data, *n* = 5,795 children, *n* = 337 taking a stimulant. (A and B) Functional connectivity (FC) difference magnitude (root-mean-square) for sleep shown on the Gordon-Laumann cortical parcellation^133^ in children (A) not taking stimulants (*n* = 5,458) and (B) taking stimulants (*n* = 337). A more liberal *t* value threshold was used in (B) to show detail. (C) Significant (FWER *p* < 0.05) differences in FC between network pairs in children not taking stimulants. (D) Magnitude (Welch’s *t*-statistic) of FC differences in whole networks, relative to the whole connectome, for sleep in children not taking stimulants and taking stimulants. Significant (FWER *p* < 0.05) changes are indicated by a *. (E) Significant (FWER *p* < 0.05) differences in FC between network pairs in children taking stimulants. DMN, default mode; VIS, visual; FPN, fronto-parietal; DAN, dorsal attention; VAN, ventral attention; SAL, salience; PMN, parietal memory; AMN, action-mode; SM, somato-cognitive action/motor; AUD, auditory; CAN, context association; HC, hippocampus; AMYG, amygdala; BG, basal ganglia; THAL, thalamus; CERB, cerebellum. See also Figures S1, S9, S11, andS12.

Longer sleep duration was associated with decreased within-network connectivity in SM, AUD, and VIS regions in children not taking stimulants (Figure 5A). Conversely, children with shorter sleep duration who were relatively sleep-deprived had increased within-network connectivity in SM, AUD, and VIS. These sleep-related differences in FC closely mirrored those in the whole cohort (Figure 3A). Remarkably, the relationship between sleep and FC vanished in the subset of children taking stimulants (see Figures 5B and S11 for the full FC matrix).

The pattern of sleep-related FC differences were parcel-wise very different in children taking a stimulant compared to children not taking a stimulant (*r* = −0.026; cortex-only *r* = 0.0004; spin test *p* = 0.997). Sleep was associated with significant (FWER *p* < 0.05) differences in SM, AUD, and VIS in children not taking stimulants (Figures 5C and 5D). There were no significant differences in FC between canonical network pairs or whole networks in the subset of children taking stimulants (Figures 5D and 5E). The edgewise sleep × stimulant interaction and the difference in sleep-related FC between children taking and not taking stimulants (Wald test)^151^ are shown in Figure S11. The difference persisted after matching for sample size (subsampling to *n* = 337 children) (Figure S11). The pattern of stimulant-related FC differences was more similar to the pattern of sleep-related FC differences in stimulant-takers with less sleep (Figure S12).

## DISCUSSION

### Stimulants modulate arousal and salience connectivity

Stimulants are one of the oldest, most potent, and most broadly used prescription psychoactive drugs, with 14 million users^1,4,5^ and over $2.2 billion annual sales in the United States,^152^ but their effects on the brain remain incompletely understood with divergent prior findings.^51^ Recent advances, including large BWAS datasets^96,125^ and PIDTs for controlled verification of BWAS findings,^92,94,129^ have allowed us to investigate the brain effects of stimulants on a scale not previously possible. Capitalizing on the recognition of somato-cognitive action regions embedded in primary sensorimotor cortex,^115^ comparison with drug target receptor maps,^64,147^ and data-driven statistical approaches^127,128^ allowed us to resolve previously ambiguous findings. This multi-modal approach revealed that the largest stimulant-related changes in FC are in somato-cognitive action and motor regions reflecting arousal state^118–120^ and in tightly coupled SAL/PMN associated with anticipation of reward/aversion and action-relevant memory.^85–87^

### Stimulants have little direct effect on attention

Prior theories regarding prescription stimulants posited direct beneficial effects on attention and control networks intersecting prefrontal cortex such as DAN, VAN (which is also a language network),^57,153^ and FPN.^21–27^ There is evidence that prefrontal cortex is associated with attention deficit in ADHD^154^ and modulated by catecholamines.^22,38,39^ However, much prior neuroimaging evidence that stimulants act primarily on attention and control networks comes from studies using ROI methodologies focused on these *a priori* networks.^24,25,27,53^ Other studies using a data-driven approach did not consistently find evidence of stimulant-related changes in canonical attention and control networks.^28,52,68^ Increased computational power, advances in statistical modeling, and large-scale datasets now enable comparison of the relative effects of stimulants on different networks with greater clarity.^102–104^ Here, with a large sample of children (*n* = 5,795), we found no significant differences in DAN, VAN, or FPN related to stimulants after accounting for larger stimulant-related differences in other brain networks (see Table 1 for a power analysis). Correspondingly, we found no significant difference in performance on the NIH Toolbox or n-back, tasks involving attention and working memory, in healthy children taking stimulants. Instead, performance of children with ADHD taking a stimulant improved to the level of the rest of the cohort. This imaging and behavioral evidence does not support the hypothesis that the primary effect of stimulants is to increase attentional ability through direct modulation of attention and control networks.

Instead, the largest stimulant-related differences in cortical FC were in somato-cognitive action and motor regions. Attempting to reconcile stimulant effects in motor cortex with their use in treating ADHD, it has been argued that stimulants might reduce motoric output by enhancing cortical inhibition in motor cortex.^88,109–114^ While inhibition of motoric output might be desirable when stimulants are taken to treat ADHD, stimulants are also effective in contexts where the goal is to increase motoric output, such as athletic enhancement.^19^ We observed that stimulant-related differences in SM FC were highly concordant with the FC pattern of getting more sleep or being more alert. Thus, the role of stimulants in SM could be related to increased sympathetic drive and higher arousal, consistent with recent insights into action and motor cortex function.^92,118–124^

The seemingly paradoxical effect that stimulants can reduce hyperactivity may instead be related to their dopaminergic effects on salience processing. The second largest stimulant-related differences in FC were in SAL/PMN, which together are thought to encode anticipated reward/aversion and thus influence the decision to persist at a task or switch to a more rewarding task.^29,59,73,74,76,77,89,155–160^ Aspects of ADHD hyperactivity could be associated with searching for more rewarding actions and thus better understood as motivational rather than motoric. We hypothesize that stimulants reduce task-switching and thus appear outwardly to facilitate attention by elevating the perceived salience of mundane tasks^42,161^ (e.g., math homework)^43,44^ through their effect on SAL, boosting persistence^34,35^ and effort^37,40,84^ without affecting cognitive ability.^34,35,40,41^ Although beyond the scope of this study, future work should assess whether stimulants increase task-fMRI activation in response to smaller anticipated rewards.

In this study, SAL and PMN were combined to address methodological limits on statistical power for networks with small areal representations^127,128^ (see network sizes in Table S4). It has recently also been suggested that SAL/PMN may be a single higher-order network.^70–80^ The joint stimulant-related differences in SAL/PMN connectivity suggest not only modulation of salience but also complementary facilitation of memory in the service of goal-directed action.^85–87^ Future studies might investigate whether stimulants improve action memory supported by the PMN and if effects on memory are dissociable from perceived salience.

### Stimulants rescue brain connectivity from short-term sleep deprivation

Stimulants increase synaptic norepinephrine,^2,3^ promoting arousal and wakefulness.^8,9,20,162,163^ We observed stimulant-related differences in sensorimotor FC aligned with norepinephrine receptor density, consistent with recent insights into somato-cognitive action and motor cortex function.^92,118–124^ Remarkably, we found that taking a stimulant before scanning made the brain connectivity of children with less sleep indistinguishable from that of well-rested children. Stimulants also rescued cognitive performance in children with less sleep. Thus, stimulants appeared to rescue the brain from the effects of sleep deprivation, at least in the short term. The ability of stimulants to rescue cognitive decrements in sleep-deprived individuals through modulation of the brain’s arousal system may be an important reason why many purported cognitive advantages of stimulants do not replicate in controlled experimental cohorts with little variation in sleep.^34,35,40,41,163^

While our results appear to show that the cognitive performance of sleep-deprived children benefited from stimulants, we caution that mounting evidence points to cumulative health consequences of long-term sleep deprivation including increased risk of depression, cellular stress, and neuronal loss.^107,164^ A washout study collecting fMRI data in sleep-deprived participants shortly after taking stimulants and later after drug levels have fallen could assess whether the beneficial effects of stimulants persist or reverse after drug concentrations taper off in the afternoon. Additional long-term studies are needed to evaluate whether stimulant users are less likely to get adequate sleep and measure the cumulative effects of sleep loss over the lifespan.

### Patients with ADHD benefit from stimulants

ADHD is the primary medical indication for stimulants.^6^ ADHD is a heterogeneous condition with reported changes in attention networks, salience networks, mixed mechanisms,^23,29,59,73,74,165,166^ and even the existence of distinct ADHD subtypes,^167–169^ including evidence from the ABCD Study.^165,166^ Our findings show that stimulants improve school grades and cognitive performance in children with ADHD without increasing cognitive ability or bestowing any unfair advantage.^17^ We also show that FC differences related to stimulants are similar to those of getting more sleep and that getting more sleep was itself associated with increased cognitive performance.^108^ Sleep disturbance is a common comorbidity of ADHD and a common complication of stimulant treatment;^170^ therefore, clinicians should screen for sleep disturbance in children with ADHD both before and after prescribing a stimulant.

### Stimulants facilitate behavior by increasing drive

Attention is a multifaceted construct that is difficult to operationalize from behavioral studies alone. Performance on attention-demanding tasks is influenced not only by cognitive ability and allocation of attention but also by arousal, vigilance, motivation, effort, and persistence or drive. Using rs-fMRI, we showed that stimulants mimic the effects of sleep (arousal) and reward expectation (salience) consistent with boosting drive,^34,35,37,40,76,84^ not top-down allocation of attention^43,44^ nor cognitive ability.^40,41^ Increased drive is consistent with the many uses of stimulants beyond the treatment of ADHD including to treat narcolepsy,^7^ promote wakefulness after TBI,^8,9^ increase diet adherence,^11–13^ and enhance athletic performance.^19^ Some of the benefits of stimulants could also be attained by getting sufficient sleep each night,^163^ something about half of children^106,107^ and adults^171^ go without. Conversely, stimulants confer little benefit in the performance of actions that are intrinsically motivating.^42–44,161^ Thus, beyond effects shared with being better rested, additional stimulant-specific effects on behavior may derive from boosting one’s drive to persist at less rewarding tasks.

### Limitations of the study

This manuscript aims to reconcile brain differences related to stimulants with their purported effect on attention, but the term “attention” is an imprecise, multifaceted concept that is difficult to operationalize and is not localized to any one brain region or network. Comparison of our findings with prior studies is complicated by limited availability of source data and the use of different parcellations and network definitions across studies. The ABCD cohort^125^ includes a mix of children taking different stimulant medications (e.g., methylphenidate, lisdexamfetamine) and diagnosed with different ADHD subtypes,^165,166^ approximating that of the United States population^172^; however, it is not powered to investigate the effects of specific medications or ADHD subtypes. Variability in scan duration and lack of precise data regarding timing and formulation (e.g., immediate vs. delayed release) of stimulant administration limit our ability to account for pharmacokinetic effects in the ABCD cohort, which could have led to an underestimation of the effect of stimulants on fMRI connectivity.^173^

## RESOURCE AVAILABILITY

### Lead contact

Further information and requests for code and data should be directed to and fulfilled by the lead contact, Benjamin Kay (benjamin.kay@wustl.edu).

### Materials availability

This study did not generate new unique reagents.

### Data and code availability

- This paper analyzes existing, publicly available data, accessible at:
  - https://doi.org/10.15154/1503209 (ABCD 2.0 release)
  - https://wustl.box.com/v/PsilocybinPFM (Precision Imaging Drug Trial)
  - https://github.com/netneurolab/hansen_receptors/ (PET receptor maps)
  - https://github.com/neurdylab/fMRIAlertnessDetection (EEG arousal template)
  - https://github.com/ryraut/arousal-waves (respiratory arousal map)
  - FC data shown in the main figures have been deposited at Zenodo and are publicly available as of the date of publication at https://doi.org/10.5281/zenodo.17916532.
- This paper does not report original code.
- Any additional information required to reanalyze the data reported in this paper is available from the lead contact upon request.

## STAR★METHODS

### EXPERIMENTAL MODEL AND STUDY PARTICIPANT DETAILS

#### ABCD participants

This project used resting-state functional MRI, demographic, biophysical, and behavioral data from 11,572 8–11 year old participants from the ABCD 2.0 release.^126^ The ABCD Study obtained centralized institutional review board (IRB) approval from the University of California, San Diego. Each of the 21 sites also obtained local IRB approval. Ethical regulations were followed during data collection and analysis. Parents or caregivers provided written informed consent, and children gave written assent. This project also includes published derivatives from other studies^119,123,124,146^ whose protocols were governed by their respective IRBs.

#### Replication in healthy adults

Five healthy adults without ADHD ages 18–45 years (2 male, 3 female) participated in a randomized cross-over pharmacometric fMRI study in which participants received methylphenidate 40 mg or psilocybin 25 mg on separate days in a random order.^129^ (A sixth participant taking a prescription stimulant was excluded from analysis.) Data from the Psilocybin PFM study^129^ were collected in accordance with protocols approved by the Washington University in St. Louis IRB. Image acquisition was divided across multiple days. Resting-state fMRI was acquired using the protocol below with multiple 15-minute-long rs-fMRI scans per day of scanning. Each participant underwent at least 4 baseline scans before receiving either methylphenidate or psilocybin; these were used as the control condition. Each participant underwent at least 2 scans 60–180 min after taking methylphenidate 40 mg by mouth. One baseline scanning session during which a participant fell asleep was excluded from analysis.

### METHOD DETAILS

#### Behavioral

The Adolescent Brain Cognitive Development (ABCD) study participants are well-phenotyped with demographic, physical, cognitive,^182^ and mental health^183^ batteries. We used the NIH Toolbox^150^ and parent reported school grades as measures of out-of-scanner cognitive ability. Data were downloaded from the NIMH Data Archive (ABCD Release 2.0), and the traits of interest were extracted using the ABCDE software we have developed and which we have made available here: https://gitlab.com/DosenbachGreene/abcde.

#### Prescription stimulant medications

The ABCD Study asked parents to recall their children’s prescription medications. Parents searched for their children’s medications on an interactive tablet linked to the RxNorm database.^184^ Parents were also asked whether their child took the medication in the last 24 h. Stimulants are dosed in the morning, therefore children whose parents reported giving stimulants within the last 24 h were assumed to have taken the stimulant on the morning of their MRI scans. Complete information about dosage and formulation (e.g., tablet, liquid, extended release) were not available for the first year of the study. Using the ABCDE software, we cross-referenced parent responses with the RxNorm database to identify children taking a drug with one of the following active ingredients: methylphenidate, dexmethylphenidate, amphetamine, dextroamphetamine, or lisdexamfetamine. The stimulant drug serdexmethylphenidate was approved by the FDA in 2021, after the first year of ABCD data had been collected. Among the sample of 5,795 children with complete data, 7.1% (73.6% boys) were prescribed a stimulant and 5.8% (73.0% boys) took the stimulant on the day of scanning (*n* = 337).

#### ADHD

Several algorithms have been proposed to identify children with ADHD in the ABCD Study.^130^ This study used the stringent “Tier 4” criteria from Cordova et al.^130^ These criteria include children who met criteria for ADHD “present” or “current” on the Kiddie Schedule for Affective Disorders and Schizophrenia (KSADS-COMP).^185^ Children with intellectual disability, bipolar disorder, schizophrenia or psychotic symptoms were excluded. Children who scored below clinical cutoff on the teacher-reported Brief Problem Monitor (BPM) scale,^186^ or who scored below clinical cutoff on the parent-reported Child Behavioral Checklist (CBCL) attention or ADHD scales^187^ were also excluded. Children with missing data were not excluded.

Among the sample of 5,795 children with complete data, 3.0% of children (66.9% boys) had ADHD of whom 43.4% were prescribed a stimulant and 34.9% took a stimulant on the day of scanning. Conversely, 18.1% of children taking a stimulant on the day of scanning had ADHD. To reconcile this paradox, we defined a less stringent criteria for ADHD used for exploratory analysis only; the stringent criteria was used for our main analyses. The less stringent criteria was based on the KSADS-COMP only and included children with ADHD “present,” “past,” “in remission,” or of an “unspecified” subtype. A majority (75.0%) of children taking stimulants met these less stringent, exploratory criteria for ADHD.

#### Sleep

Parents were asked questions about their child’s sleep disturbances.^140^ We reversed the order of the responses to create a monotonically increasing scale of average sleep duration with 1 = less than 5 h, 2 = 5–7 h, 3 = 7–8 h, 4 = 8–9 h, and 5 = greater than 9 h. We used average sleep duration as a surrogate measure of arousal/wakefulness at the time of scanning.

#### Covariates

Following published guidelines for the ABCD Study,^188,189^ we selected average in-scanner head motion (framewise displacement, FD), age (in months), sex (assigned at birth), household income bracket, highest level of education achieved by a parent, and whether or not parents were married as nuisance covariates. The marriage covariate was supplemented by an additional covariate describing whether there was one or more than one adult caregiver in the household (regardless of marital status). There is controversy regarding inclusion of race or genetic ancestry as a default covariate^189^; we did not include race as there is no biologically plausible mechanism by which it would affect the brain’s response to stimulant medications.

We selected additional covariates relevant to our hypotheses, including ADHD diagnosis (using the stringent “Tier 4” criteria above).^130^ Diurnal variations are reported to affect FC,^190^ therefore we also included time of scan (morning or afternoon), and day of week (weekday or weekend). Except where otherwise noted, sleep duration was included as a covariate in analyses of stimulants, and stimulant taking was included as a covariate in analyses of sleep duration.

Modeling of linear covariates does not guarantee that variation in sample size and demographic characteristics related to taking stimulants have been adequately controlled for.^191^ Therefore, we performed a supplemental analysis using a cohort of *n* = 337 children not taking stimulants and not diagnosed with ADHD most closely matching the demographic characteristics *n* = 337 children taking stimulants on the day of scanning using MATLAB’s^180^ knnsearch (Figure S2).

#### ABCD MR imaging

Functional magnetic resonance imaging (fMRI) was acquired at 21 sites using a protocol harmonized for 3 Tesla GE, Philips, and Siemens scanners with multi-channel receive coils.^135^ In addition to anatomical and task-fMRI, each participant had up to four 5-minute-long resting-state scans (TR = 800 ms, 20 min total). A subset of sites using Siemens scanners used FIRMM motion tracking software that allows extending the scan on the basis of on-line measurement of motion.^192^

Following acquisition, fMRI data were processed using standardized methods including correction for field distortion, frame-by-frame motion co-registration, alignment to standard stereo-tactic space, and extraction of the cortical ribbon.^193^ Resting-state data were further processed to remove respiratory and motion artifact by temporal bandpass filtering, global signal regression, and regression against the rigid-body motion parameters using the ABCD-BIDS motion processing pipeline,^175^ a derivative of the Human Connectome Project (HCP) processing pipeline,^194^ and utilities from the ABCD-BIDS Community Collection (ABCC).^176^ Processing dependencies included FSL,^177^ FreeSurfer,^178^ and NiBabel.^181^ Functional MRI data acquired at different study sites were harmonized using CovBat.^195–197^

#### Healthy adult MR imaging

The fMRI acquisition protocol was similar to ABCD. We used an echo-planar imaging sequence with 2 mm isotropic voxels, multiband 6, multi-echo 5 (TEs: 14.20 ms, 38.93 ms, 63.66 ms, 88.39 ms, 113.12 ms), TR 1761 ms, flip angle = 68°, and in-plane acceleration (IPAT/grappa) = 2. This sequence acquired 72 axial slices (144 mm coverage). Each resting scan included 510 frames (lasting 15:49 min) as well as 3 frames at the end used to estimate electronic noise. Data were processed using a previously-described custom pipeline^129^ including thermal noise removal using NORDIC,^198^ correction of slice timing and field distortions, motion co-registration, optimal combination of echos by weighted summation,^199^ intensity normalization, non-linear registration to the MNI atlas, bandpass filtering, and motion censoring at a framewise displacement (FD) of 0.2 mm. We modified the pipeline to perform global signal regression to more closely match the ABCD data.

Data were co-registered to the same atlas as the ABCD data and parcellated using the same 394 parcellation used for the ABCD data. A 394 × 394 FC matrix was computed for each rs-fMRI scan using the methods above and motion censoring threshold of FD < 0.2 mm as in the ABCD data. An edge-wise linear mixed effects model was used to compare scans on methylphenidate to baseline scans. The data on psilocybin were not used. Sex was modeled as a fixed effect. The model included a random intercept for scan session (a day of scanning) as well as a random intercept and slope (for methylphenidate) within participant. Due to the small number of participants (*n* = 5), we did not attempt to perform permutation-based significance testing or network level analysis. Edge-wise *t*-values are reported in Figure S8 and were used to generate the cortical surface maps shown in Figure 2.

#### Parcellation

It is possible to compute functional connectivity between each voxel or vertex. However, this approach is burdened by a high proportion of unstructured noise and large computer memory requirements. We therefore adopted a parcel-based approach based on the 333 cortical parcels described by Gordon and Laumann^133^ augmented by the 61 subcortical spheres described by Seitzman^200^ for a total of 394 parcels, or nodes.

#### Removing head motion artifact

Motion in fMRI studies is typically estimated using spatial co-registration of each fMRI volume (or frame) to a reference frame.^201^ In this study we quantified motion using framewise displacement, FD (L1-norm), in millimeters, after filtering for respiratory artifact.^175,202^ Exclusion of frames with FD > 0.2 mm has been shown to reduce spurious findings associated with residual motion artifact in high-motion groups,^174,203^ such as children with ADHD. Participants with less than 8 min (600 frames) of resting-state data remaining after motion censoring, the minimum duration needed for high-quality estimation of connectivity,^91^ were excluded from analysis. Of the 11,875 children recruited in the first wave of the ABCD Study, 8,486 had more than 8 min of rs-fMRI data after censoring frames with FD > 0.2 mm.

#### Motion impact assessment

After removing head motion artifact, we quantified the impact of residual head motion artifact on our brain-behavior associations of interest, stimulant and sleep duration, using the SHAMAN method.^174^ The covariates described above were included as regressors of non-interest.

#### Functional connectivity

We employed standard approaches for computing functional connectivity. The methods are briefly summarized here. By convention, each brain region or parcel is referred to as a node. The functional connections between nodes, which are referred to as edges, are computed as the pairwise linear correlation coefficients between nodes. As correlations are constrained to vary from −1 to 1, the correlation coefficients were Fisher Z transformed (inverse hyperbolic tangent function) to lie on an approximately normal distribution. Ordinary least squares (OLS) regression was performed independently at each edge.

#### Generation of brain maps

Analysis of rs-fMRI data was performed on (394^2^-394)/2 = 77,421 distinct edges arising from the 333 Gordon-Laumann cortical parcels and 61 Seitzmann subcortical spheres.^133,200^ Some results were projected back into the space of the 333 cortical parcels for visualization as brain maps. Seed-based FC maps were generated from an exemplar seed parcel in somatomotor hand region, which was selected *a posteriori* as the parcel with the greatest difference in FC related to stimulants. Brain maps of magnitude difference in FC were generated by computing the root-mean-square (RMS) average change in FC for each row in the FC matrix. The RMS values were rendered on their corresponding cortical parcels using Connectome Workbench.^179^

In Figure S3 the vertex-wise nucleus accumbens seed map was generated using the subcortical volume for nucleus accumbens from the Human Connectome Project.^144,194^ In Figure S12, the value at each cortical parcel was computed as the linear (Pearson) correlation between each row in the stimulant FC matrix with each corresponding row in the sleep FC matrix.

#### Norepinephrine transporter data

PET maps were compiled by Hansen et al.^147^ and projected onto the cortical surface using Connectome Workbench.^179^ The map of norepinephrine transporter was generated using the 11C-MRB (methylreboxetine) ligand (*n* = 20).^146^ The supplemental dopamine receptor map for D1 was generated using the 11C-SCH23390 ligand (*n* = 13).^204^ The D2 map was generated using the 11C-FLB457 ligand (*n* = 6).^205^

The data were downloaded from: https://github.com/netneurolab/hansen_receptors.

#### Independent arousal data

The ABCD Study does not include physiologic arousal data, therefore we compared FC differences related to sleep duration (a surrogate measure of arousal) in ABCD data to physiologic arousal maps from two independent studies (Figure 4).

- The EEG alpha slow wave index arousal template (*n* = 10)^123,124^ was projected onto the cortical surface using Connectome Workbench.^179^

The data were downloaded from: https://github.com/neurdylab/fMRIAlertnessDetection.

- The respiratory variation arousal map (see Figure S2B “PLV_magnitude” from Raut et al.)^119^ was generated from *n* = 190 participants with simultaneous fMRI and respiratory (chest bellows) data in the WU-Minn Human Connectome Project (HCP) 1200 Subject Release.^144^

The data were downloaded from: https://github.com/ryraut/arousal-waves.

The arousal map is found under: output_files/HCP_RV_coherencemap.dtseries.nii.

### QUANTIFICATION AND STATISTICAL ANALYSIS

#### Marginal model and bootstrapping

The ABCD data are clustered by study site and family (some participants are siblings). In addition to data harmonization across sites with CovBat,^195–197^ we explicitly modeled site differences and sibling relationships in our statistical analyses. A linear mixed-effects model with site and family random effects would have been computationally expensive due to the large number of participants and features (edges) in this study. Instead, we employed a marginal model in which the fixed effects beta-values are obtained through ordinary least squares regression. The standard error is corrected for non-exchangeability of the residuals due to clustering by site and family using the Huber-White sandwich estimator.^206,207^ The marginal beta values are divided by the corrected standard errors to obtain cluster-robust marginal *t*-values corrected for site and family. Edgewise statistical inference was performed using wild bootstrap under the null model with the Rademacher distribution.^208^ This approach has been shown to yield comparable results to mixed effects regression at lower computational cost in large neuroimaging datasets.^176,209^

#### Network level analysis

We are principally interested in FC differences involving canonical networks (e.g., DMN, VIS, etc.), not differences involving specific edges. Network Level Analysis (NLA) is an adaptation of enrichment analysis that performs inference at the level of canonical networks. We performed NLA using previously described methods.^127,128^ Briefly, FC values at each edge were studentized using the cluster-robust sandwich estimator approach described above^206,207^ to obtain edge-level FC *t*-values. The average FC *t*-value of edges within each network pair (e.g., DMN and VIS) was compared with the average FC *t*-value value over the whole connectome using Welch’s *t* test.^210^ A Welch’s *t*-value of zero indicated no difference in FC relative to the whole connectome. A positive Welch’s *t*-value indicated enrichment of FC differences within a network pair, i.e., a large change in connectivity. A negative Welch’s *t*-value indicated depletion of FC differences within a network pair, i.e., a small change in connectivity.

Separately, whole networks were compared to the connectome by averaging the absolute values of the FC *t*-values in each network. Positive Welch’s *t*-values indicated enrichment of FC differences within a network, i.e., a large change in connectivity.

Inference was performed by generating a null distribution of Welch’s *t*-values using the same wild bootstrap procedure described above^208^ with 2,000 bootstrap iterations. The Westfall-Young step-down procedure^134^ was used to control the family wise error rate (FWER) from comparisons across multiple network pairs.

NLA is biased toward detection of significant changes in large networks pairs with many edges. Therefore, related networks with a small number of nodes were combined as indicated in Figure S1 and Table S4 for the purpose of statistical inference. Lateral and medial visual networks were combined into a single visual network. Salience and parietal memory networks were combined into a single SAL/PMN network. Premotor, somatomotor hand, somatomotor mouth, somatomotor foot, and somatocognitive action networks were combined into a single SM network.

#### Power analyses

Like all methods to account for multiple statistical comparisons, network level analysis (NLA) controls the false positive error rate at the expense of false negative error rate, or statistical power. A prior study^52^ on stimulant-related FC differences within attention networks with *n* = 24 participants reported a *t*-value of 4.35 corresponding to an effect size (Cohen’s *d*) of 0.89. We assessed the power of our NLA approach to detect an FC difference of this size within attention or control networks: DAN, VAN, or FPN. For each network, we simulated a Welch’s *t*-value using the formula:

$$t_{\text{net}}=\frac{\frac{d}{\sigma_{\text{stim}}}-\bar{t}}{\sqrt{\frac{\sigma_{t}}{n_{\text{tot}}}+\frac{\sigma_{t}}{n_{\text{net}}}}}$$

Where.

- *t*_net_ is the Welch’s *t*-statistic for a network
- *d* is the effect size, e.g., 0.89
- *σ*_stim_ = 0.058 is the standard error of regression for stimulants,

i.e., the square root of the diagonal element in (*X*^⊤^*X*)^−1^

- $\bar{t}$ = 0.031 is the average *t*-statistic for all edges in the connectome
- *σ*_*t*_ = 1.35 is the standard deviation of *t*-statistics in the connectome
- *n*_tot_ = 77,421 is the total number of edges in the connectome
- *n*_net_ is the number of within-network edges (DAN: 496, VAN: 253, FPN: 276)

The simulated Welch’s *t*-value, *t*_net_, was ranked against the bootstrapped null distribution of Welch’s *t*-values to compute a *p*-value. The *p*-value was corrected for multiple comparisons using the Westfall-Young step-down procedure. Power to detect an effect size *d* within the given network was calculated as 1 - *P*. See Table 1 for minimum detectable effect sizes at different power levels.

#### Statistical comparison of brain maps

Inference on similarity between brain maps was performed using the rotational null model of Vázquez-Rodríguez for parcellated surface maps^136,211^ using the NeuroMaps software.^137^ The Vázquez-Rodríguez model accounts for the medial wall of the cortical surface by reassigning missing data to the nearest parcel.^136,137^ Comparisons were performed for the 333 parcels on the cortical surface^133^ only. Many maps were thresholded at 50% intensity for visual presentation (e.g., Figure 4), but the whole range of intensity values were used for quantifying similarity. First we calculated the real correlation *r* between two maps across the 333 parcels. Then we generated 2,000 rotational null maps and computed the correlations *r*_Ø,1_,… *r*_Ø,2000_ between each pair of null maps. Finally, we computed the *p*-value for the two-tailed alternative hypothesis of *r ≠* 0 by counting the number of permutations in which |*r*| < |*r*_Ø_| and dividing by the total number of permutations.

#### Task-fMRI analysis

The n-back task was used in the ABCD Study to engage working memory and cognitive control in adolescents.^135^ There was less fMRI data available for the n-back task compared to rest due to greater scan time allocated to resting-state data acquisition; consequently, there were only *n* = 1,944 children with high-quality n-back data (FD < 0.2 mm and greater than 8 minutes of scan time) of whom *n* = 109 took a stimulant on the day of scanning. N-back data were analyzed in two ways. First, data were treated as rest, without regressing out the task paradigm, to test the hypothesis that stimulants would affect FC during an attention-demanding task differnetly than they would at rest, (Figure S4). Second, we performed conventional task-fMRI analysis for the 0-back and 2-back vs. fixation contrast using FSL’s FEAT with default settings^177,212,213^ (Figure S4).

## Supplementary Material

Supplemental Tables

1

Supplemental information can be found online at https://doi.org/10.1016/j.cell.2025.11.039.

## Figures and Tables

**Table 1.** Power analyses

Power level	Effect Size		
	DAN	VAN	FPN
95%	0.13	0.17	0.17
80%	0.10	0.14	0.14

Minimum detectable effect size (Cohen’s *d*) at different power levels for stimulant-related differences in functional connectivity (FC) within attention and control networks. Effect sizes are for statistical inference with network level analysis (NLA). Previously reported effect sizes for stimulant-related FC differences in attention networks are approximately *d* = 0.89.^52^ NLA was at least 95% powered to detect FC differences of this size. DAN, dorsal attention network; VAN, ventral attention network; FPN, frontoparietal network.

**Table 2.** Differences in cognitive performance related to ADHD, stimulants, and sleep

Measure	ADHD			Stimulant			Sleep		
	Effect	SE	*p* value	Effect	SE	*p* value	Effect	SE	*p* value
School Grade	−0.82	0.068	1.2 × 10^−32^	0.28	0.019	0.154	0.096	0.013	2.143 × 10^−13^
NIH Toolbox	−5.57	1.05	1.3 × 10^−7^	0.087	3.04	0.98	0.40	0.20	0.045
N-Back Correct	−0.054	0.014	8.7 × 10^−5^	−0.017	0.037	0.64	0.013	0.0025	1.9 × 10^−7^
N-Back RT	−0.64	12.4	0.96	−101	33.5	0.0025	2.18	2.27	0.33

Measure	ADHD × Stimulant			Stimulant × (−Sleep)		
	Effect	SE	*p* value	Effect	SE	*p* value
School Grade	0.34	0.12	0.0057	0.10	0.045	0.025
NIH Toolbox	8.00	1.89	2.4 × 10^−5^	0.70	0.70	0.32
N-Back Correct	0.050	0.024	0.039	−0.0011	0.0086	0.90
N-Back RT	−19.7	21.604	0.36	−20.492	7.76	0.00083

A linear regression model was used to predict school letter grade (1 = F, 5 = A), NIH Toolbox score (mean = 50, SD = 10),^150^ n-back correct response rate (1 = 100% correct), and n-back reaction time (RT, in milliseconds) from ADHD diagnosis and sleep duration (hours) with sex, age, and socioeconomic factors as covariates in *n* = 5,795 children, *n* = 337 taking stimulants. ADHD and sleep were each associated with significant improvements on cognitive performance, while stimulants were observed to most improve performance for children with ADHD (ADHD × stimulant interaction) or sleep deprivation (stimulant × −sleep interaction). SE, standard error. Standardized effect sizes and Cohen’s *d* are reported in Table S3.

**Table:** KEY RESOURCES TABLE

REAGENT or RESOURCE	SOURCE	IDENTIFIER
Deposited data		
Adolescent brain cognitive development (ABCD)	Jernigan,^126^ Casey et al.^135^	https://doi.org/10.15154/1503209; https://abcdstudy.org/scientists/data-sharing/
Precision Imaging Drug Trial	Siegel et al.^129^	https://wustl.box.com/v/PsilocybinPFM
Positron Emission Tomography	Hansen et al.^147^	https://github.com/netneurolab/hansen_receptors/
EEG Arousal	Falahpour et al.,^123^ Goodale et al.^124^	https://github.com/neurdylab/fMRIAlertnessDetection
Respiratory Variation Arousal	Rautetal.^119^	https://github.com/ryraut/arousal-waves
Software and algorithms		
Motion Impact Score (SHAMAN)	Kay et al.^174^	https://github.com/DosenbachGreene/shaman
Network Level Analysis (NLA)	Li etal.^128^	https://github.com/WheelockLab/MachineLearning_NetworkLevelAnalysisBeta
NeuroMaps	Markello et al.^137^	https://github.com/netneurolab/neuromaps
ABCD-HCP Pipeline (DCAN-BOLD)	Fair etal.,^175^ Feczko et al.^176^	https://github.com/DCAN-Labs/abcd-hcp-pipeline
FMRIB Software Library (FSL)	Jenkinson et al.^177^	https://fsl.fmrib.ox.ac.uk
Freesurfer	Fischl et al.^178^	https://surfer.nmr.mgh.harvard.edu/
Connectome Workbench	Marcus et al.^179^	https://www.humanconnectome.org/software/connectome-workbench
MATLAB	Mathworks^180^	https://www.mathworks.com/
NiBabel	Brett et al.^181^	https://github.com/nipy/nibabel/

## Highlights

- Stimulants altered functional connectivity in action regions consistent with arousal
- Stimulants altered functional connectivity in salience regions consistent with reward
- Stimulants did not affect canonical attention networks
- Stimulants reversed the behavioral and brain effects of sleeping less
