# Supplementary material for: Stimulant medications affect arousal and reward, not attention networks
Source: Cell. Author manuscript; Available in PMC 2026 Jan 26. (PMC12834599; doi:10.1016/j.cell.2025.11.039)
Supplement: Supplemental Tables [file NIHMS2132972-supplement-Supplemental_Tables.pdf]

Cell, Volume *188*

Supplemental information

Stimulant medications affect arousal and reward, not attention networks

Benjamin P. Kay, Muriah D. Wheelock, Joshua S. Siegel, Ryan V. Raut, Roselyne J. Chauvin, Athanasia Metoki, Aishwarya Rajesh, Andrew Eck, Jim Pollaro, Anxu Wang, Vahdeta Suljic, Babatunde Adeyemo, Noah J. Baden, Kristen M. Scheidter, Julia S. Monk, Forrest I. Whiting, Nadeshka Ramirez-Perez, Samuel R. Krimmel, Russell T. Shinohara, Brenden Tervo-Clemmens, Robert J.M. Hermosillo, Steven M. Nelson, Timothy J. Hendrickson, Thomas Madison, Lucille A. Moore, Óscar Miranda-Domínguez, Anita Randolph, Eric Feczko, Jarod L. Roland, Ginger E. Nicol, Timothy O. Laumann, Scott Marek, Evan M. Gordon, Marcus E. Raichle, Deanna M. Barch, Damien A. Fair, and Nico U.F. Dosenbach

	Overestimation		Underestimation	
Variable	Z-score	P-value	Z-score	P-value
Stimulant	-4.97	0.890	10.5	0.085
Sleep	-13.4	0.963	30.0	0.006

Supplemental Table 1: **Motion impact scores, related to Figures 1 and 3.** An assessment of residual head motion artifact[174] after motion censoring at FD < 0.2 mm was performed for stimulants and sleep duration. Significant motion overestimation scores indicate a risk of detecting spurious FC differences. Significant motion underestimation scores indicate a risk of failing to detect FC differences. Both stimulants and sleep had not-significant motion overestimation scores ($P > 0.05$, uncorrected), indicating an acceptably low risk of spurious findings due to head motion. Sample size n = 5,795. See also Supplemental Figure 7.

	Stimulant		Sleep Duration	
	Correlation	P-value	Correlation	P-value
Norepinephrine	0.36	0.0125	0.32	0.005
EEG Alpha/Delta	0.37	0.0085	0.49	< 0.0001
Respiratory Variation	0.41	0.0225	0.51	0.0015

Supplemental Table 2: **Similarity of sleep and arousal brain maps, related to Figures 1, and 3.** The ABCD Study included n = 5,795 children, 337 taking a stimulant. The norepinephrine transporter binding map was derived from positron emission tomography (PET) data using 11C-MRB (methylreboxetine) in n = 20 participants.[145,146] The EEG alpha/delta power ratio (alpha slow wave index) map was derived from n = 10 participants.[123,124] The respiratory variation map was generated using data from n = 190 participants from the Human Connectome Project.[119] Correlations are reported for 333 cortical parcels.[173] Significance testing was performed using spin tests.[135,136] See also Figure 4.

	ADHD			Stimulant			Sleep		
Measure	Effect	SE	Cohen's d	Effect	SE	Cohen's d	Effect	SE	Cohen's d
School Grade	**-0.82**	-0.16	-1.09	-0.16	0.019	0.045	**0.077**	0.010	0.10
NIH Toolbox	**-5.57**	1.05	-0.51	-2.94	0.69	-0.27	**0.32**	0.16	0.030
N-Back Correct	**-0.054**	0.014	-0.40	-0.013	0.0086	-0.095	**0.011**	0.0020	0.079
N-Back RT	-0.64	12.4	-0.0056	-12.8	7.7	-0.11	1.76	1.82	0.015
	ADHD × Stimulant						Stimulant × (-Sleep)		
Measure	Effect	SE	Cohen's d				Effect	SE	Cohen's d
School Grade	**0.34**	0.12	0.45				**0.081**	0.036	0.11
NIH Toolbox	**8.00**	1.89	0.74				0.56	0.57	0.052
N-Back Correct	**0.050**	0.024	0.37				-0.0009	0.0069	0.0065
N-Back RT	-19.7	21.6	-0.17				**-16.5**	6.24	-0.14

Supplemental Table 3: **Standardized differences in cognitive performance related to ADHD, stimulants, and sleep, related to Table 2.** A linear regression model was used to predict school letter grade (1 = F, 5 = A), NIH Toolbox score (mean 50, SD 10), [149] n-back correct response rate (1 = 100% correct), and n-back reaction time (RT, in milliseconds) from ADHD diagnosis and sleep duration (hours) with sex, age, and socioeconomic factors as covariates in n = 5,795 children, 337 taking stimulants. P-values are identical to Table 2. Significant ($P < 0.05$) effects are bolded. In this table, the effect size has been standardized by centering each predictor variable and scaling it by its standard deviation. Cohen's d was computed by dividing the standardized effect size by the standard deviation of the dependent variable (e.g. grade). SE = standard error.

Network		Nodes
Default Mode	DMN	41
Visual	VIS	33
Medial Visual	MEDVIS	6
Frontoparietal	FPN	24
Dorsal Attention	DAN	32
Ventral Attention	VAN	23
Salience	SAL	4
Parietal Memory	PMN	5
Action Mode	AMN	40
Premotor	PREMOT	9
Somatomotor Hand	SMH	14
Somatomotor Mouth	SMM	6
Somatomotor Foot	SMF	13
Somatocognitive Action	SCAN	4
Auditory	AUD	24
Context Association	CAN	8
Hippocampus	HC	4
Amygdala	AMYG	2
Basal Ganglia	BG	16
Thalamus	THAL	12
Cerebellum	CERB	27

Supplemental Table 4: **Network size, related to Network level analysis in the STAR Methods.** The name and size (number of nodes) of each canonical network is shown along with the grouping of smaller, functionally-related networks into larger networks for the purpose of network level analysis (NLA). For locations of networks see Supplemental Figure 1.

	Stimulant		Sleep Duration	
Receptor/Transporter	Pearson	Spearman	Pearson	Spearman
Norepinephrine	0.3623	0.3050	0.3177	0.2835
Dopamine D1	-0.2756	-0.2251	-0.1629	-0.1296
Dopamine D2	-0.0316	0.0216	-0.0027	0.0149

Supplemental Table 5: **Correlation of FC differences with monoaminergic receptor/transporter densities, related to Figure 4.** Parcellated cortical receptor densities were obtained from positron emission tomography (PET) studies,[146] see Figure 10. Stimulant- and sleep-related differences in FC were strongly correlated with norepinephrine transporter density and weakly correlated with dopamine receptor density. Norepinephrine transporter (NET) maps were generated using the 11C-MRB ligand ($n = 20$).[145] D1 receptor maps were generated using the 11C-SCH23390 ligand ($n = 13$).[204] D2 receptor maps were generated using the 11C-FLB457 ligand ($n = 6$).[205] See also Figure 4 and Supplemental Figure 10.
