# Supplementary material for: Stimulant medications affect arousal and reward, not attention networks
Source: Cell. Author manuscript; Available in PMC 2026 Jan 26. (PMC12834599; doi:10.1016/j.cell.2025.11.039)
Supplement: 1 [file NIHMS2132972-supplement-1.pdf]

Supplemental figures

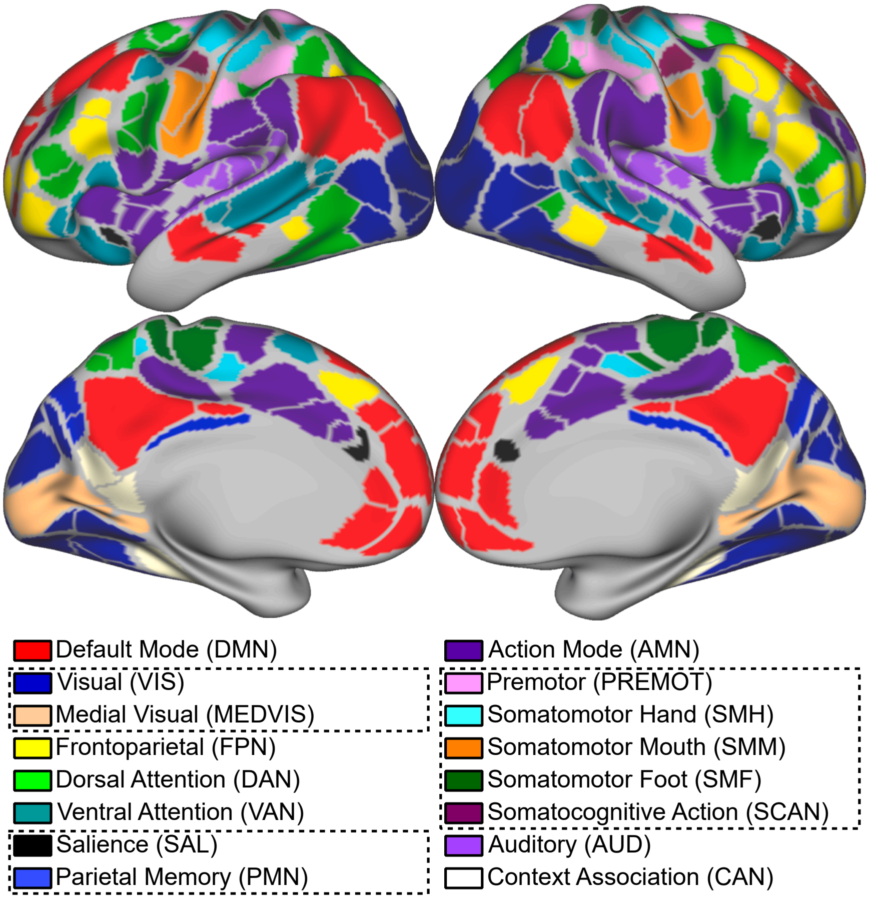

Figure S1. Network communities shown on the Gordon-Laumann 333 cortical parcels, related to Table 1 and Figures 1, 3, and 5

Networks grouped together for network level analysis are indicated by boxes.[133]

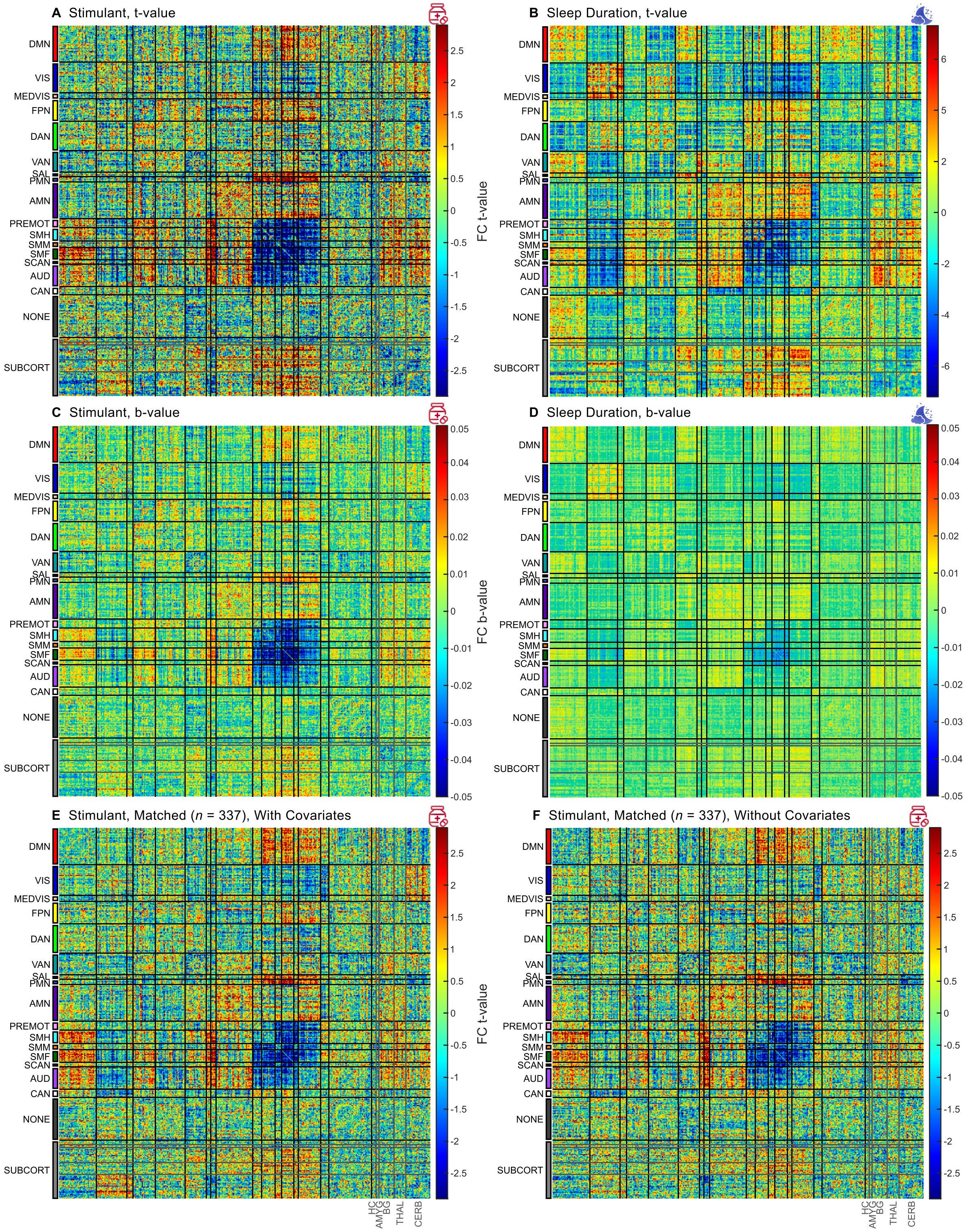

Figure S2. Differences in FC related to stimulants and sleep, related to Figures 1 and 3

(A) Differences in FC related to taking a stimulant on the day of scanning (n = 5,795, n = 337 taking stimulants). The t value and effect size (beta value) are shown.

(B) Differences in FC related to sleep duration. The FC matrices were edge-for-edge correlated at r = 0.38.

(C and D) Difference in FC related to stimulants and sleep rendered as raw beta values (regression weights).

(legend continued on next page)

(E) Differences in FC related to taking a stimulant in n = 337 children compared to a matched cohort of n = 337 children not taking a stimulant, accounting for demographic covariates. The FC matrix was edge-for-edge correlated with FC for the full cohort at r = 0.78.

(F) Differences in stimulant-related FC in the matched cohort, modeled without covariates. The FC matrix was edge-for-edge correlated with FC for the full cohort at r = 0.75.

For names and locations of networks, see Figure S1.

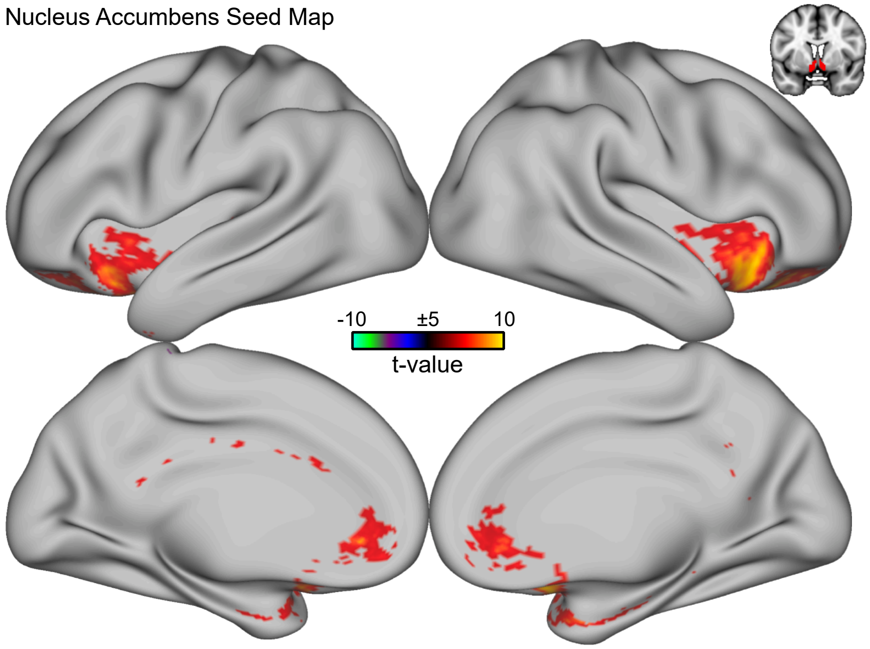

Figure S3. Nucleus accumbens seed map, related to Figure 1
FC (*t*-value) between nucleus accumbens and cortex is shown for all *n* = 5,795 participants in ABCD. Accumbens FC overlaps with right anterior inferior insula, the hub of the salience network. The nucleus accumbens seed is shown at top right. There was no significant difference in accumbens-cortex FC related to taking stimulants.

CellPress
OPEN ACCESS

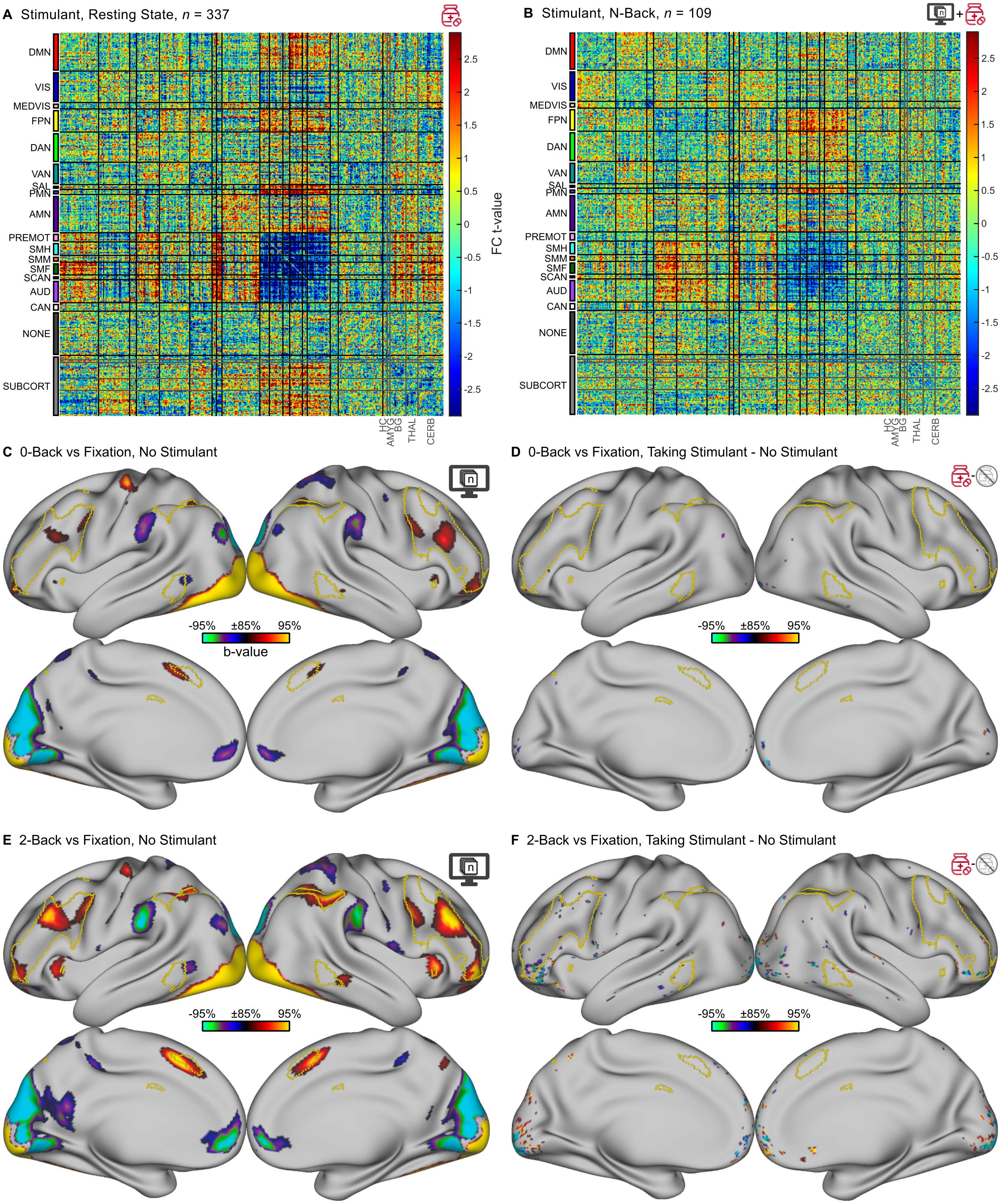

Figure S4. Influence of the n-back task, related to Figure 1

(A) Stimulant-related differences in FC during resting state with n = 337 participants taking stimulants among a total of n = 5,795 participants, as in Figure S2.

(B) Stimulant-related differences in data collected during the n-back task treated as rest with n = 109 children taking stimulants. There were less fMRI data available for the n-back task compared to rest due to greater scan time allocated to resting-state data acquisition; consequently, there were only n = 1,944 children with high-quality n-back data. The task paradigm was not regressed out. The FC matrices were edge-for-edge correlated at r = 0.26.

(legend continued on next page)

(C) Task-evoked activation for 0-back vs. fixation contrast in all participants. Regression coefficients (beta-values) between 85% and 95% of the maximum are shown. The frontoparietal network (FPN) is outlined in yellow.
(D) Higher-order contrast for 0-back vs. fixation in children taking stimulants (*n* = 109) vs. not taking stimulants, shown on the same beta-value scale.
(E) 2-back vs. fixation.
(F) Children taking stimulants vs. not taking stimulants, 2-back vs. fixation.
For names and locations of networks, see Figure S1.

CellPress
OPEN ACCESS

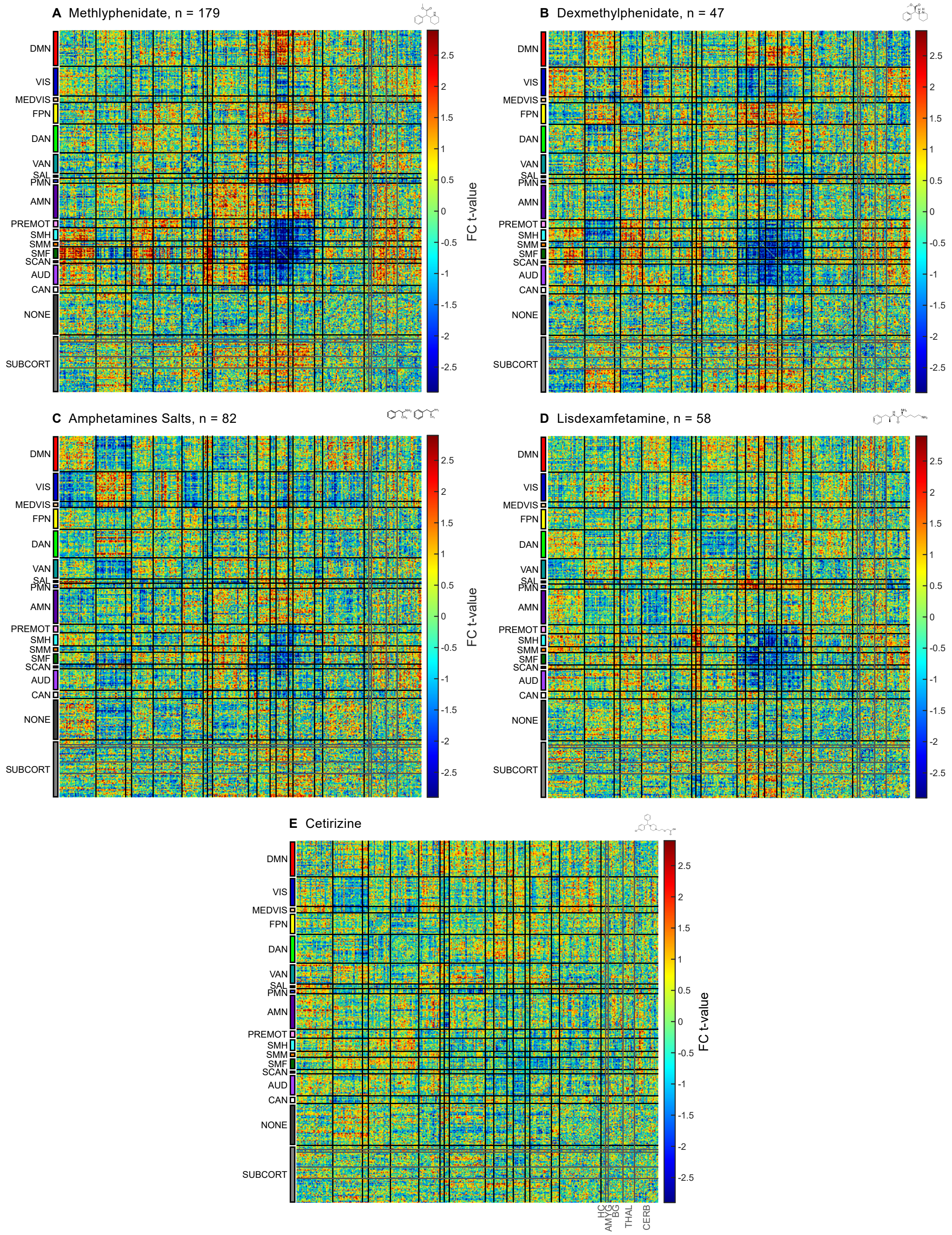

Figure S5. Differences in FC related to different stimulant drugs, related to Figure 1

(A) Methylphenidate (Ritalin), $n = 179$ children. The FC matrix for methylphenidate was edge-for-edge correlated with the pooled FC matrix for all stimulants at $r = 0.81$.

(B) Dexmethylphenidate (Focalin), $n = 47$, $r = 0.53$.

(C) Mixed amphetamine salts (Adderall), $n = 82$, $r = 0.54$.

(D) Lisdexamfetamine (Vyvanse), $n = 58$, $r = 0.48$.

(E) Cetirizine, a commonly taken allergy medication without psychoactive properties,[139] was selected as a negative control. The FC matrix for cetirizine is edge-for-edge correlated with that of stimulants at $r = 0.10$. Total $n = 5{,}795$, $n = 291$ taking cetirizine within 24 h before scanning.

For names and locations of networks, see Figure S1.

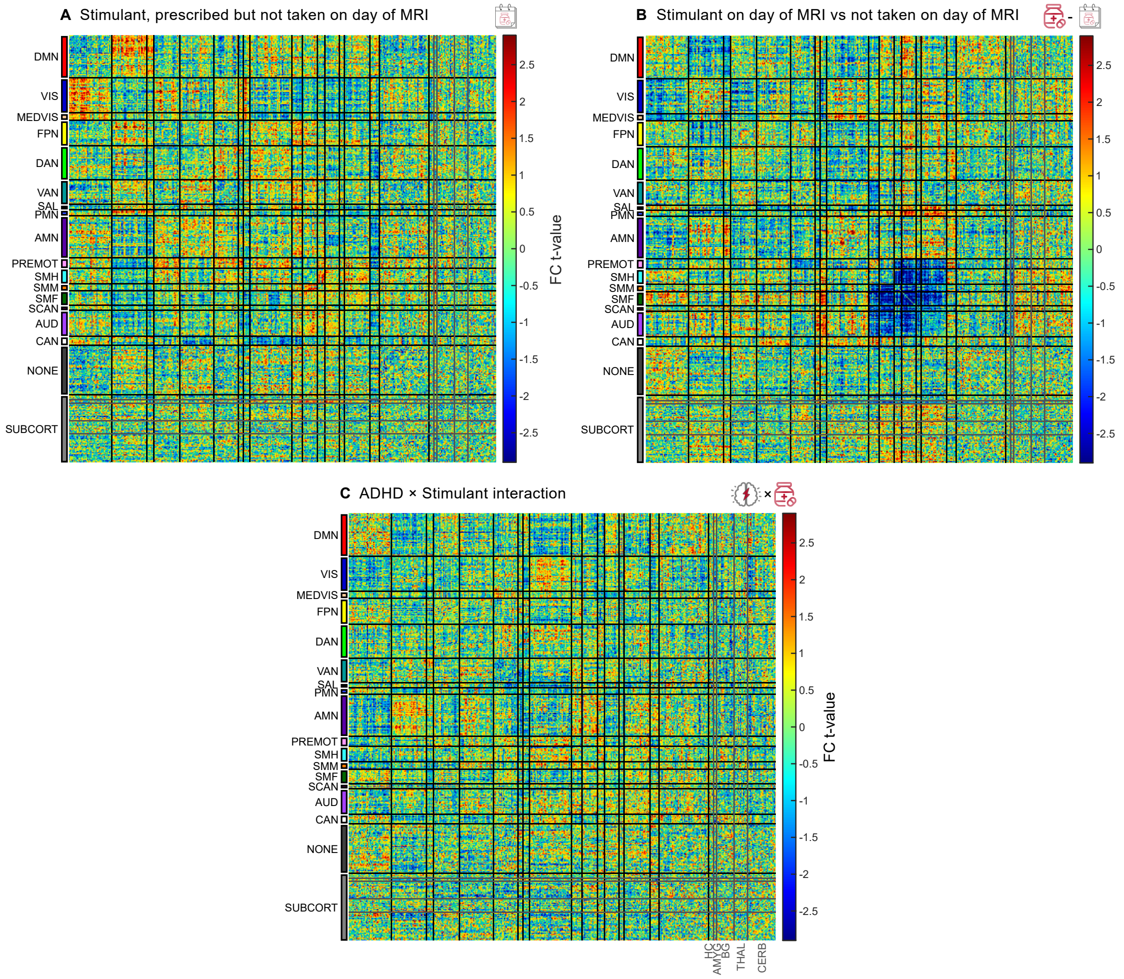

Figure S6. Differences in FC related to stimulants not taken on the day of scanning and to ADHD, related to Figure 1

(A) Difference between $n = 76$ children who were prescribed a stimulant but did not take it on the day of scanning and $n = 5,382$ children not prescribed a stimulant. The FC matrix was edge-for-edge correlated with that of stimulants taken on the day of scanning vs. all other participants (Figure S2) at $r = 0.015$.

(B) Difference between $n = 337$ children who took a stimulant on the day of scanning and $n = 76$ children prescribed a stimulant who did not take it on the day of scanning. The FC matrix was edge-for-edge correlated with that of stimulants taken on the day of scanning vs. all other participants at $r = 0.55$.

(C) ADHD-specific differences in FC are shown for an edgewise linear model of stimulant × ADHD interaction with sex, ADHD, and stimulant as covariates. Total $n = 5,795$, $n = 337$ taking stimulants, $n = 195$ with ADHD, $n = 67$ with ADHD taking stimulants.

For names and locations of networks, see Figure S1.

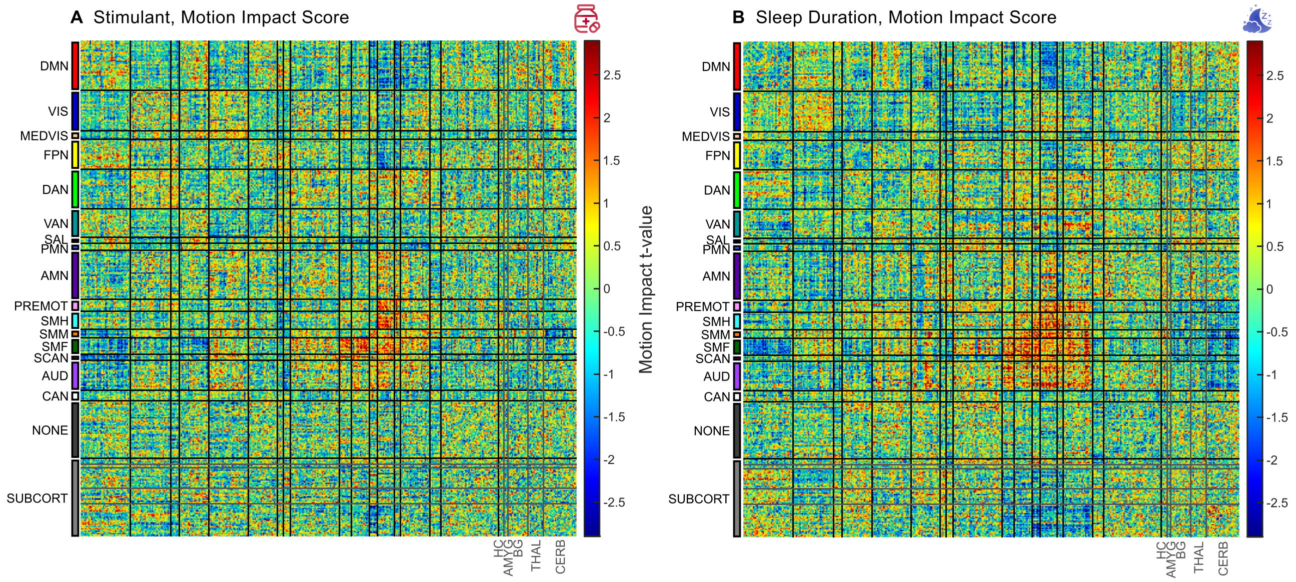

Figure S7. Motion impact assessment, related to Figures 1 and 3

Data were motion censored at framewise displacement (FD) < 0.2 mm. Motion impact scores reveal the effect of residual head motion artifact on stimulant- and sleep-related FC differences.[174] Motion impact scores were anticorrelated with stimulant- and sleep-related FC differences; therefore, the risk of motion-induced spurious findings is low.

(A) Motion impact score for stimulants (n = 5,795, n = 337 taking stimulants).

(B) Motion impact score for sleep duration (n = 5,795).

For names and locations of networks, see Figure S1.

See also Table 1.

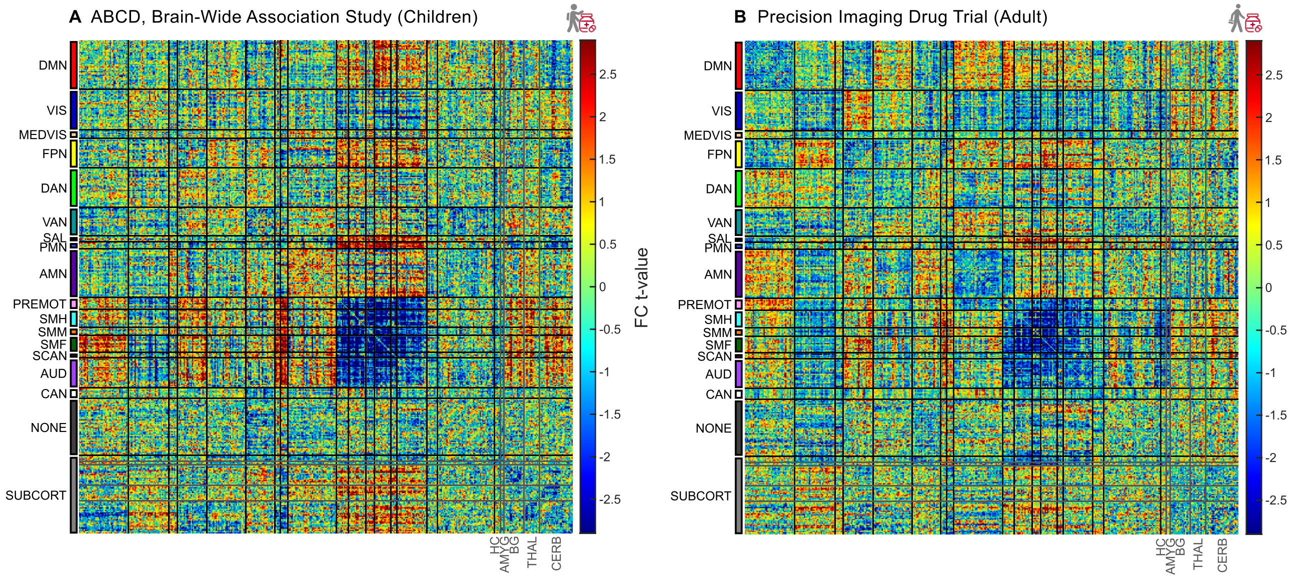

Figure S8. Comparison of stimulant-related FC differences across studies, related to Figures 1 and 2

(A) Children in the ABCD Study (*n* = 337 taking stimulant, *n* = 5,795 total).

(B) Adults without ADHD in a controlled methylphenidate drug trial (*n* = 5).[129] The FC matrices are edge-for-edge correlated at *r* = 0.19.

For names and locations of networks, see Figure S1.

CellPress
OPEN ACCESS

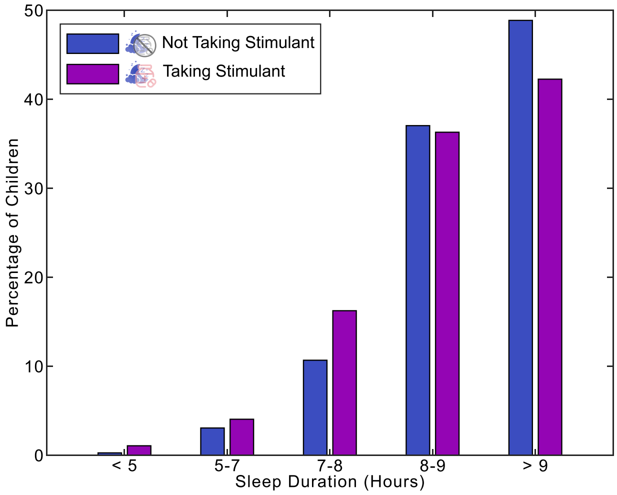

Figure S9. Sleep duration (in hours), related to Figures 3 and 5

Parent-reported average sleep duration is shown for children who did and did not take a stimulant on the day of scanning. For the purpose of reporting effect sizes, we treated one ordinal unit of sleep duration as approximately equal to one hour (60 min) of sleep. Children who took a stimulant on the day of scanning got 10 fewer minutes of sleep per night (n = 5,795, n = 337 on stimulants, $p = 1.0 \times 10^{-4}$). After controlling for age, sex, demographic covariates, and ADHD (tier 4 criteria),[130] the effect shrank to 3.1 min (p = 0.23). ADHD diagnosis was associated with 14.8 fewer minutes of sleep per night (n = 175 with ADHD, $p = 3.2 \times 10^{-5}$).

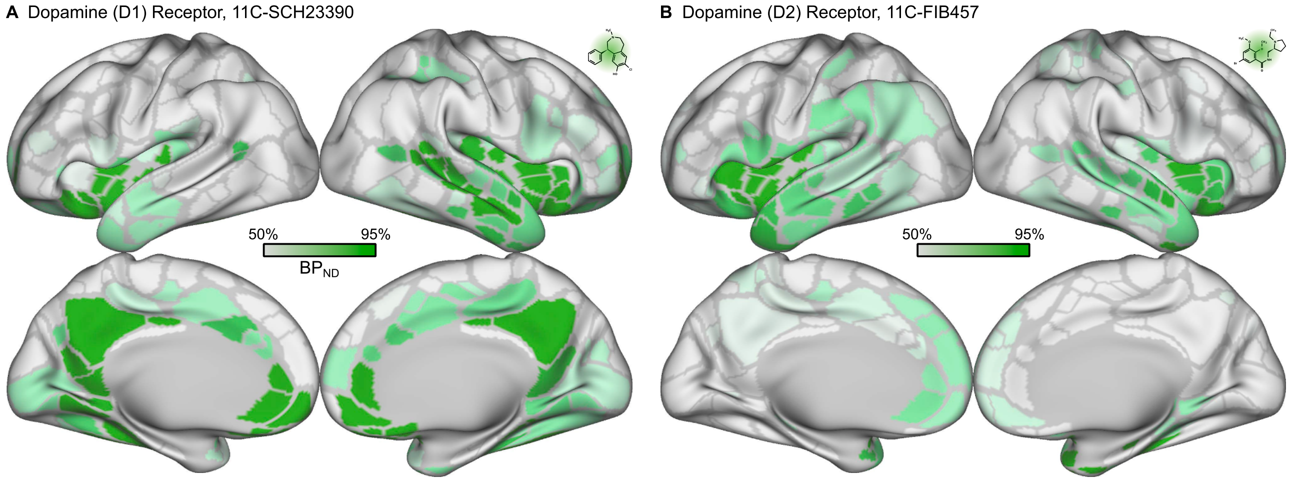

Figure S10. Dopamine receptor maps, related to Figure 4

Parcellated cortical receptor densities were obtained from positron emission tomography (PET) studies.[147]

(A) D1 receptor maps were generated using the 11C-SCH23390 ligand (*n* = 13).[204]

(B) D2 receptor maps were generated using the 11C-FLB457 ligand (*n* = 6).[205]

See also Table S5.

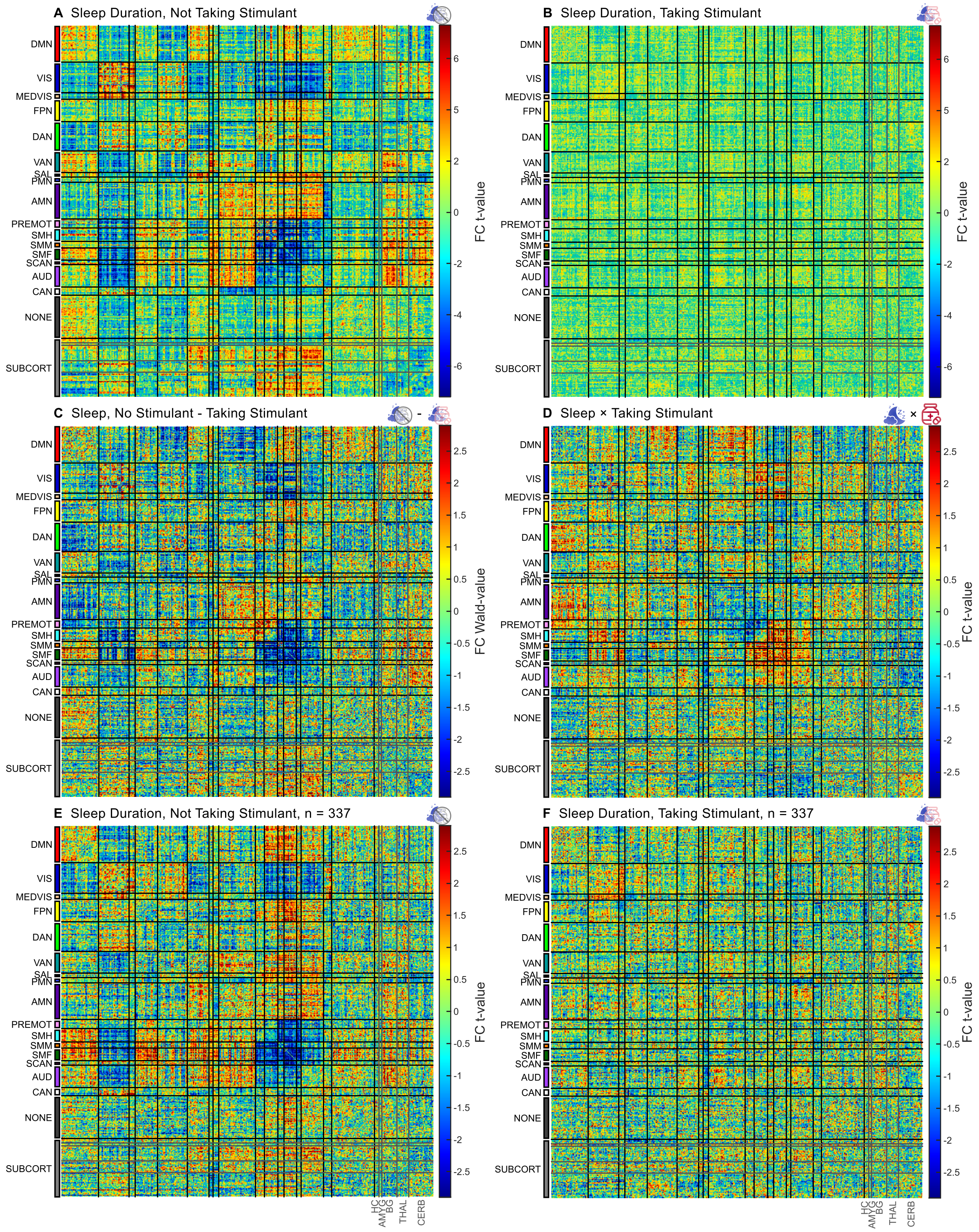

Figure S11. Differences in FC related to sleep in children on and off stimulants, related to Figure 5

(A) Children not taking a stimulant (n = 5,458).

(B) Children taking a stimulant on the day of scanning (n = 337).

(C) Wald test, sleep in children not taking stimulants minus sleep in children taking stimulants (n = 337).

(D) Sleep × stimulant interaction.

(legend continued on next page)

(E) Children not taking a stimulant (matched sample size *n* = 337).
(F) Children taking a stimulant (*n* = 337), visually matched color scale.
For names and locations of networks, see Figure S1.

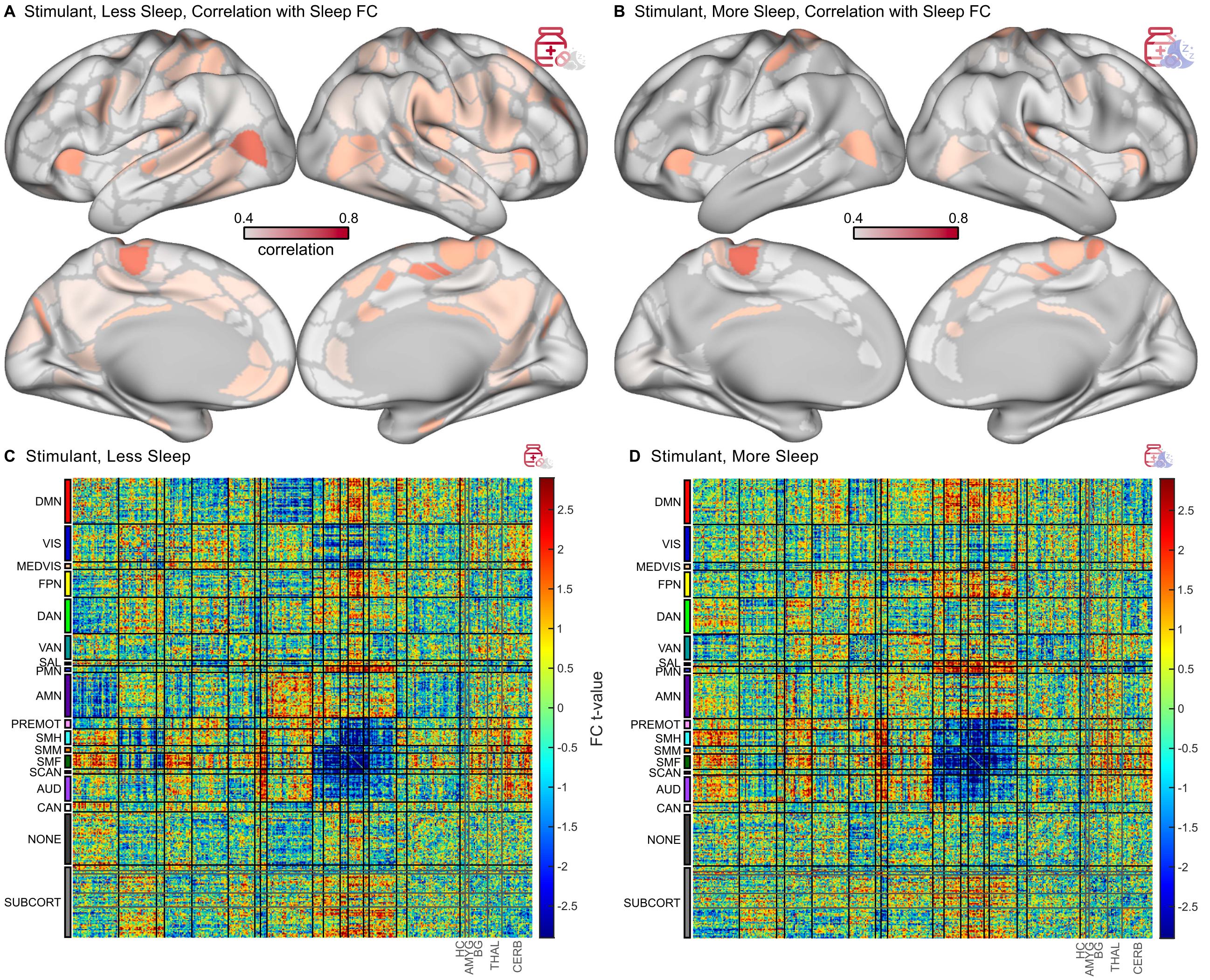

Figure S12. Relationship between stimulants and sleep, related to Figure 5

FC differences related to stimulants were compared to FC differences related to sleep. The edgewise correlation between FC differences related to stimulants and sleep is shown for each cortical parcel. Negative values of correlation are shown in gray.

(A) Children with less than 8 h of sleep (*n* = 804, *n* = 68 taking stimulants).

(B) Children with more than 8 h of sleep (*n* = 2,883, *n* = 148 taking stimulants). The FC differences related to stimulants and sleep were more similar in children getting less sleep.

(C and D) Children getting less than 8 h of sleep (C) (*n* = 804, *n* = 68 taking stimulants) and children getting more than 8 h of sleep (D) (*n* = 2,883, *n* = 148 taking stimulants).

For names and locations of networks, see Figure S1.
